# Association of oxidized ApoB and oxidized ApoA-I with high-risk coronary plaque features in cardiovascular disease

**DOI:** 10.1172/jci.insight.172893

**Published:** 2023-10-23

**Authors:** Alexander V. Sorokin, Christin G. Hong, Angel M. Aponte, Elizabeth M. Florida, Jingrong Tang, Nidhi Patel, Irina N. Baranova, Haiou Li, Philip M. Parel, Vicky Chen, Sierra R. Wilson, Emily L. Ongstad, Anna Collén, Martin P. Playford, Thomas L. Eggerman, Marcus Y. Chen, Kazuhiko Kotani, Alexander V. Bocharov, Alan T. Remaley

**Affiliations:** 1 Section of Inflammation and Cardiometabolic Diseases, Cardiovascular Branch,; 2 Proteomics Core, and; 3 Section of Lipoprotein Metabolism, Translational Vascular Medicine Branch, National Heart, Lung, and Blood Institute, NIH, Bethesda, Maryland, USA.; 4 Department of Laboratory Medicine, Clinical Center, NIH, Bethesda, Maryland, USA.; 5 Bioinformatics/Integrated Data Sciences Section, Research Technology Branch, National Institute of Allergy and Infectious Diseases, NIH, Bethesda, Maryland, USA.; 6 Bioscience Cardiovascular, Research and Early Development, and; 7 Projects, Research and Early Development, Cardiovascular, Renal, and Metabolism, BioPharmaceuticals R&D, AstraZeneca, Gaithersburg, Maryland, USA.; 8 Division of Community and Family Medicine, Jichi Medical University, Shimotsuke, Tochigi, Japan.

**Keywords:** Cardiology, Inflammation, Atherosclerosis, Cardiovascular disease, Lipoproteins

## Abstract

**BACKGROUND.:**

Oxidized apolipoprotein B (oxLDL) and oxidized ApoA-I (oxHDL) are proatherogenic. Their prognostic value for assessing high-risk plaques by coronary computed tomography angiography (CCTA) is missing.

**METHODS.:**

In a prospective, observational study, 306 participants with cardiovascular disease (CVD) had extensive lipoprotein profiling. Proteomics analysis was performed on isolated oxHDL, and atherosclerotic plaque assessment was accomplished by quantitative CCTA.

**RESULTS.:**

Patients were predominantly White, overweight men (58.5%) on statin therapy (43.5%). Increase in LDL-C, ApoB, small dense LDL-C (*P* < 0.001 for all), triglycerides (*P* = 0.03), and lower HDL function were observed in the high oxLDL group. High oxLDL associated with necrotic burden (NB; β = 0.20; *P* < 0.0001) and fibrofatty burden (FFB; β = 0.15; *P* = 0.001) after multivariate adjustment. Low oxHDL had a significant reverse association with these plaque characteristics. Plasma oxHDL levels better predicted NB and FFB after adjustment (OR, 2.22; 95% CI, 1.27–3.88, and OR, 2.80; 95% CI, 1.71–4.58) compared with oxLDL and HDL-C. Interestingly, oxHDL associated with fibrous burden (FB) change over 3.3 years (β = 0.535; *P* = 0.033) when compared with oxLDL. Combined Met136 mono-oxidation and Trp132 dioxidation of HDL showed evident association with coronary artery calcium score (*r* = 0.786; *P* < 0.001) and FB (*r* = 0.539; *P* = 0.012) in high oxHDL, whereas Met136 mono-oxidation significantly associated with vulnerable plaque in low oxHDL.

**CONCLUSION.:**

Our findings suggest that the investigated oxidized lipids are associated with high-risk coronary plaque features and progression over time in patients with CVD.

**TRIAL REGISTRATION.:**

ClinicalTrials.gov NCT01621594.

**FUNDING.:**

National Heart, Lung, and Blood Institute at the NIH Intramural Research Program.

## Graphical abstract

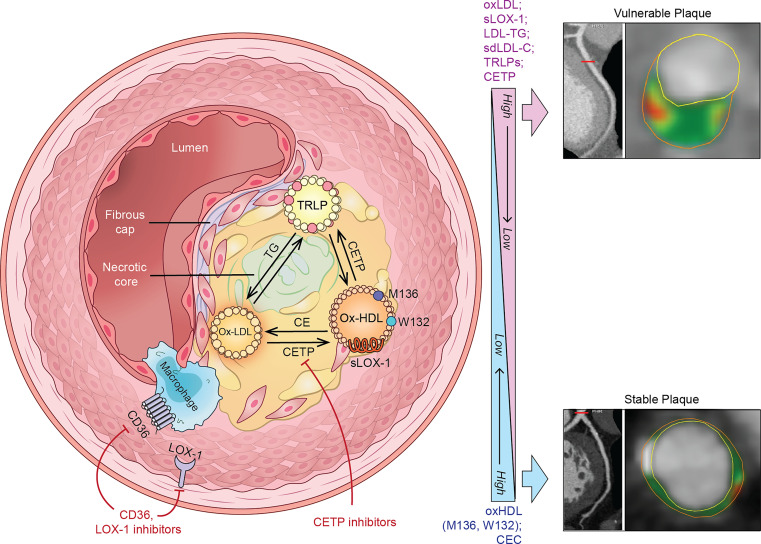

## Introduction

Persistent inflammation and dyslipidemia are well-established risk factors contributing to atherosclerosis pathogenesis (1). The cholesterol content of low-density lipoproteins (LDL-C) has been the main lipid metric that is used clinically for assessing cardiovascular disease (CVD) risk (2). Recently, there has been a growing appreciation of the possible use of alternative markers of LDL, such as ApoB (3), LDL particle number (4), LDL subfractions such as small dense LDL-C (sdLDL-C) (5), and related lipoproteins like Lp(a) (6), in the management of patients for CVD risk reduction. LDL is also known to undergo modifications, such as oxidation (7), and oxidized LDL (oxLDL/oxApoB) has also been proposed as a potential risk marker for CVD (8).

Oxidized lipoproteins can be classified as damage-associated molecular patterns, which can directly stimulate inflammation and are also avidly taken up by scavenger receptors on macrophages in the vessel wall. One of these macrophage scavenger or pattern recognition receptors, namely CD36, has been established as an emerging marker of cardiometabolic diseases, including myocardial infarction (9) and type 2 diabetes mellitus (10). Another receptor that can bind to oxLDL is lectin-type oxLDL receptor 1 (LOX-1), which triggers inflammation and release of the extracellular domain of this receptor called soluble LOX-1 (sLOX-1). Plasma levels of sLOX-1 have been reported to be significantly increased in acute myocardial infarction patients (11) and are associated with vulnerable atherosclerotic coronary plaques (12).

In contrast to LDL, elevated ApoA-I–containing high-density lipoprotein (HDL) particles are usually inversely related to CVD risk. The cholesterol content of HDL (HDL-C) is routinely used as a negative risk factor, but, like LDL-C, it has limitations, and very high levels of HDL-C may not be protective (13). Measures of HDL function such as cholesterol efflux capacity (CEC) have been clinically proven to be more sensitive and predictive of future CVD events (14). Furthermore, the function of HDL is contingent on its composition and particle size, which also determine its pleiotropic effects, including antioxidative capacity. Similarly to LDL, HDL can also undergo oxidation, which interferes with its activity like CEC (15) and also its interaction with cholesteryl ester transfer protein (CETP) (16). Dysfunctional HDL, due to oxidation or nonenzymatic glycation, has shown a positive relationship with increased CVD risk (17) and coronary artery disease (CAD). Some reports observed a strong association between oxidized HDL (oxHDL/oxApoA-I) plasma levels with atherosclerotic plaque characteristics (18) and coronary artery calcium (CAC) progression (19). Notably, most of these clinical studies had a limited number of enrolled participants and differing methods for measuring oxHDL. Indeed, the term “dysfunctional HDL” does not provide a clear definition of structural and compositional changes of the HDL/ApoA-I particles; thus our group uses the term “oxidation-modified lipoproteins” (OMLs) to better describe such modification (20). Another challenge is that the functional properties of oxHDL seem to differ depending on what specific amino acid residues in ApoA-I are targeted by either myeloperoxidase or malondialdehyde (21).

Thus, we aimed in the present study to investigate the relationship between OMLs, both those related to LDL and HDL, and high-risk features of coronary atherosclerotic plaques, as assessed by quantitative coronary computed tomography angiography (CCTA) in a cohort of patients with CVD.

## Results

### Characteristics of the study and control cohorts.

Enrolled patients (*n* = 306) had a diagnosis of CAD in 91.5% of cases and were predominantly White men (58.5%) with a BMI of 28.41 ± 6.65 kg/m^2^, and 43.5% were on statin therapy (Supplemental Table 1; supplemental material available online with this article; https://doi.org/10.1172/jci.insight.172893DS1). Although the comparative control cohort was matched by sex, it was represented by relatively younger healthy Japanese (100.0%) individuals with lower BMI of 23.15 ± 3.16 kg/m^2^ and higher total cholesterol (TC) and LDL-C. Both oxLDL (88.29 U/L [IQR, 62.15–116.06] vs. 49.20 U/L [IQR, 41.8–59.97]) and oxHDL (646.49 U/L [IQR, 442.29–864.19] vs. 135.96 U/L [IQR, 85.1–191.16]) were significantly higher in the study group as compared with control participants (*P* < 0.001 for all) (Supplemental Table 1).

When the study cohort was stratified by 50th percentile oxLDL plasma values, the high oxLDL group was characterized by younger obese participants with a lower rate of statin therapy as compared with the low oxLDL group (Table 1). These differences were accompanied by significant increases in TC, LDL-C, ApoB (*P* < 0.001 for all), and triglycerides (TGs) (*P* = 0.03) in comparison with patients with lower oxLDL. In addition, TG-rich LDL (LDL-TG) and sdLDL-C plasma levels were markedly higher as compared with the low oxLDL group (16.86 ± 6.26 mg/dL vs. 14.42 ± 4.25 mg/dL and 32.1 [25–45.4] mg/dL vs. 26.1 [18.6–33.8] mg/dL, respectively; *P* < 0.001). NMR lipid profile revealed an overall increase in LDL particles and TG-rich lipoproteins (TRLPs). Specifically, total oxLDL was correlated with small LDL (0.221) and very small TRLP (0.223) particles (*P* = 0.0002 for all) and negatively with large HDL particles (–0.175; *P* = 0.003) (Supplemental Table 2). Effects of statin therapy on some of the observed lipid changes are presented in Supplemental Tables 3 and 4. Moreover, patients with higher oxLDL levels had significantly increased markers of systemic inflammation (high-sensitivity C-reactive protein [hsCRP] and GlycA), as well as less efficient HDL function, as measured by CEC (1.04 ± 0.25 vs. 1.07 ± 0.21; *P* = 0.08) and CETP activity (4.51 [3.31–6.09] pmol/μL/h vs. 5.21 [3.96–6.44] pmol/μL/h; *P* = 0.03) (Table 1). Finally, coronary plaque burdens and CCTA vulnerable plaque characteristics were significantly higher in patients with CVD with elevated oxLDL.

Stratification of the cohort by 50th percentile oxHDL plasma values revealed opposite effects under high oxHDL levels. As a result, patients with high oxHDL were older and had a higher rate of statin treatment as compared with their counterparts in the low oxHDL group (Table 2). Changes in lipid profile were characterized by significantly higher HDL-C and ApoA-I (*P* < 0.001 for all) with a decrease in TGs (*P* = 0.02) in comparison with the patients with lower oxHDL. NMR lipid profile showed overall increase in HDL particles and a significant decrease in TRLPs, along with lower small LDL levels and higher large HDL particle levels. CETP activity was significantly decreased in patients with high oxHDL as compared with the low-oxHDL counterparts (5.07 [3.77–6.83] pmol/μL/h vs. 4.11 [3.12–5.62] pmol/μL/h; *P* = 0.01). There were no differences in CEC, oxLDL, and soluble CD36 (sCD36) between the groups; however, sLOX-1 levels were significantly reduced in high-oxHDL participants compared with low-oxHDL (228 [157–289] pg/mL vs. 171 [132–264] pg/mL, respectively; *P* = 0.04) (Table 2). Lastly, patients with CVD with high oxHDL plasma levels had significantly lower vulnerable plaque characteristics, fibrofatty burden (FFB), and necrotic burden (NB) (*P* < 0.001 for all) and increased CAC score when compared with the low oxHDL group (0 [0–4.03] vs. 2.74 [0–5.21]; *P* = 0.02).

### Association between CCTA plaque characteristics and OMLs.

**Figure 1:**
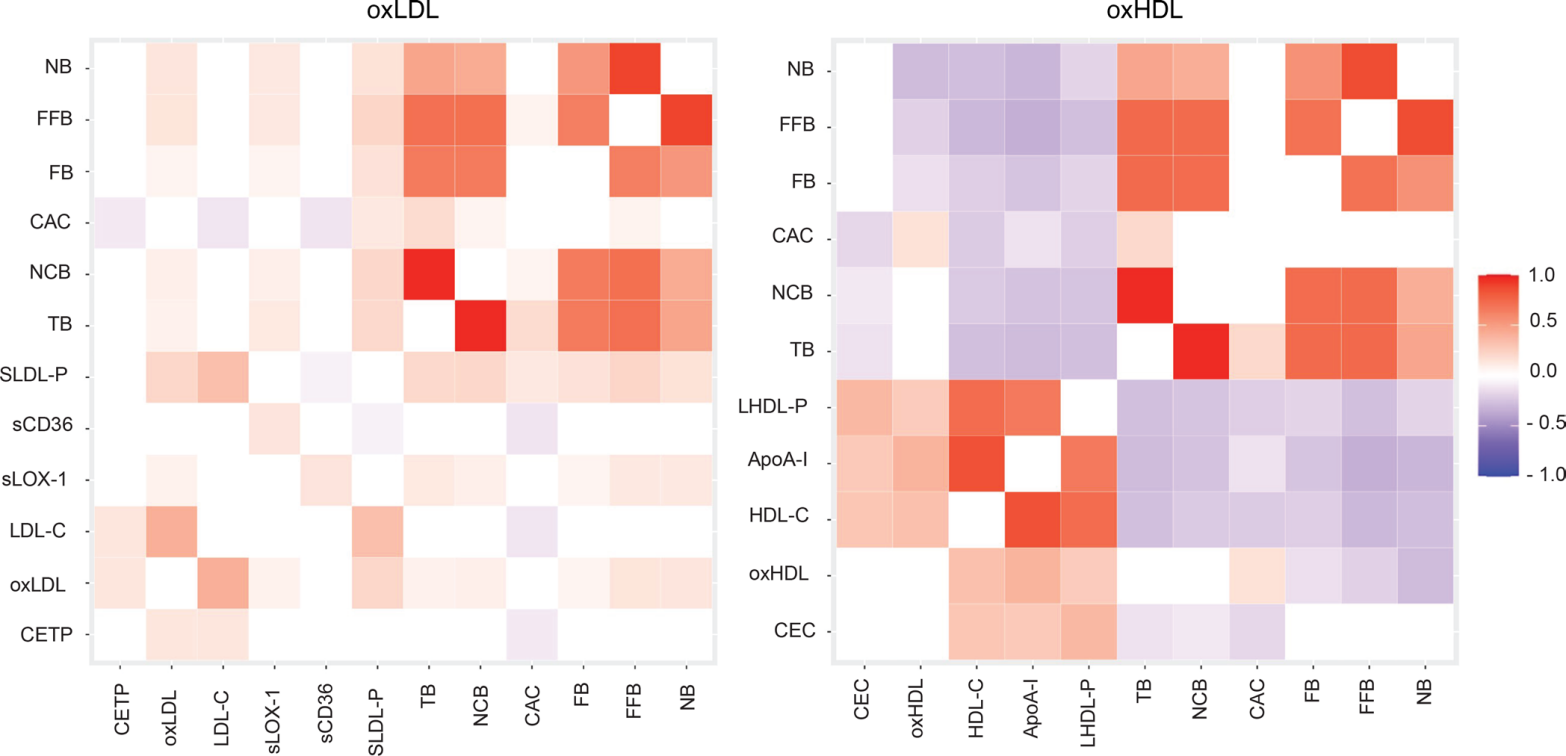
Comparison of associations of oxLDL, oxHDL, and other lipoproteins with CCTA plaque characteristics. Heatmap for Spearman’s correlation coefficients reported as *r* coefficient (*P* values). *P* < 0.05 was considered significant. CAC, coronary artery calcium (Agatston score); CEC, cholesterol efflux capacity; CETP, cholesteryl ester transfer protein; FB, fibrous plaque burden; FFB, fibrofatty burden; NB, necrotic burden; NCB, noncalcified burden; oxHDL, oxidized HDL; oxLDL, oxidized LDL; sCD36, soluble CD36; SLDL-P, small LDL particle; sLOX-1, soluble LOX-1; TB, total burden.

When oxLDL was compared with other lipoproteins, significant positive correlation with coronary plaque characteristics was observed for both oxLDL and sLOX-1 (Figure 1 and Supplemental Table 5A). Interestingly, direct LDL-C and sdLDL-C did not establish any noticeable association with the CCTA high-risk coronary plaque features, whereas small LDL particles had the most significant association with all coronary plaque variables among the lipid tests measured. Moreover, CETP activity showed only significant negative correlation with CAC (–0.092; *P* = 0.007). Further univariate adjustment revealed significant positive association of oxLDL with total plaque burden (TB) and noncalcified plaque burden (NCB) in the high oxLDL stratum (Supplemental Table 6A), which became even more evident for FFB (β = 0.15; *P* = 0.001) and NB (β = 0.20; *P* < 0.0001) after multivariate adjustment (Supplemental Table 6B).

Comparison of oxHDL with other lipoproteins and HDL function markers revealed results opposite to the oxLDL findings. A significant inverse association of oxHDL with coronary plaque vulnerable characteristics was observed along with a positive correlation with the CAC score (*r* = 0.149; *P* = 0.049) (Figure 1 and Supplemental Table 7B). Overall, ApoA-I showed the most prominent inverse association with all coronary plaque variables, whereas CEC was significantly correlated only with coronary plaque burdens and CAC score. Univariate analysis showed high oxHDL negative association with TB after BMI adjustment (β = –0.12; *P* = 0.04) and positive correlation of total oxHDL with CAC score after adjustment for sex (β = 0.18; *P* = 0.02) and TGs (β = 0.15; *P* = 0.05) (Supplemental Table 7A). Multivariate adjustment showed inverse association of high and total oxHDL with fibrous plaque burden (FB), which remained significant for total oxHDL and FFB (β = –0.14; *P* < 0.003) (Supplemental Table 7B). Moreover, low oxHDL had a tendency for negative association with NB, which became more significant after full adjustment in the total oxHDL group (β = –0.25; *P* < 0.0001). Overall comparison between OMLs found high oxLDL to be positively associated with FFB and NB, whereas low oxHDL had reverse association with these plaque characteristics (Supplemental Table 8).

**Figure 2:**
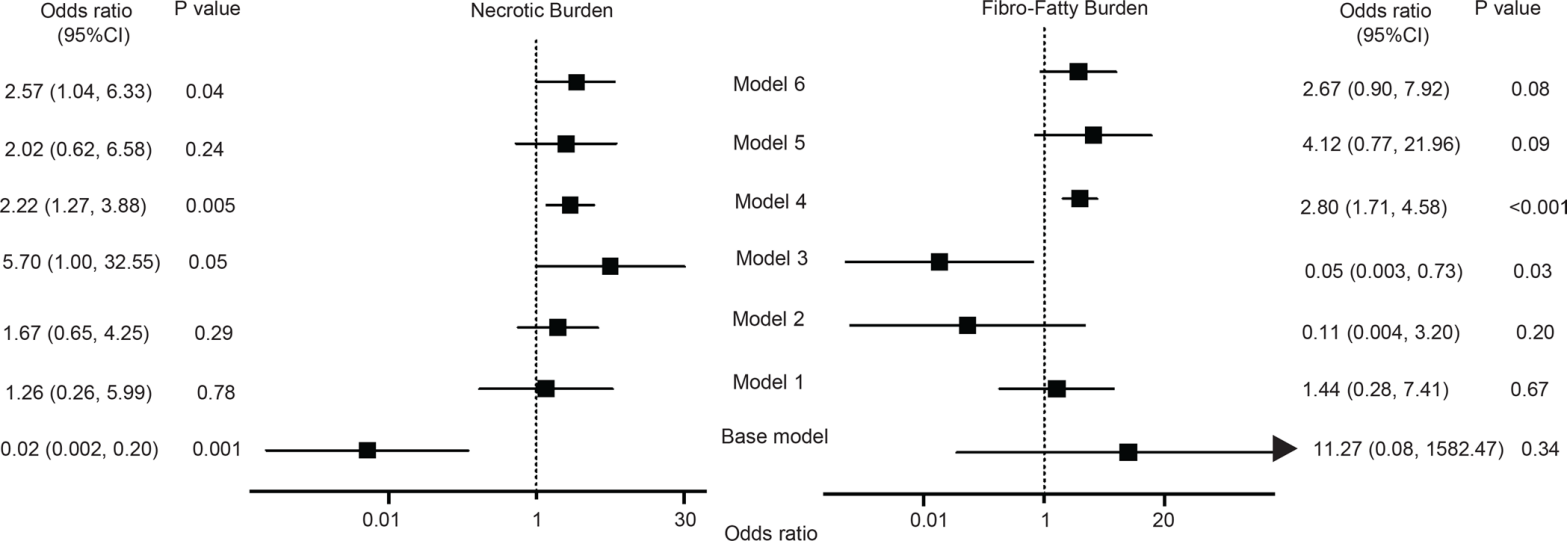
Odds ratios (95% CIs) of investigated lipids and high-risk coronary plaque characteristics. *P* < 0.05 was considered significant. Base model: adjusted for age, sex, current smoking, BMI, hsCRP, and statin treatment. Model 1: base model + LDL-C. Model 2: base model + HDL-C. Model 3: base model + oxLDL. Model 4: base model + oxHDL. Model 5: base model + sCD36. Model 6: base model + sLOX-1. Necrotic burden and fibrofatty burden were log-transformed.

Considering that both oxLDL and oxHDL had evident differences in their relation to CCTA plaque morphology, we performed logistic regression modeling (ORs, 95% CIs) to further define which of the oxidized markers have better prediction of plaque vulnerability parameters beyond traditional CVD risk factors. OxHDL had a better predictive value for NB over oxLDL, when added to the base model adjusted for traditional risk factors, including statin treatment and hsCRP (2.22, 1.27–3.88, vs. 5.70, 1.00–32.55) (Figure 2). Interestingly, oxHDL was more specific toward NB, as well as for FFB, and it predicted better than oxLDL after use of the same base model (2.80, 1.71–4.58, vs. 0.05, 0.003–0.73) (Figure 2). Finally, oxHDL was significantly associated with FB change over 3.3 years follow-up in a limited number of enrolled patients with CVD (ρ = 0.535; *P* = 0.033) when compared with oxLDL (ρ = –0.041; *P* = 0.841).

### Functional properties of OxHDL and its association with lipids of interest.

Considering that oxidation of HDL results in structural and functional changes, we aimed to understand how ApoA-I–containing lipoproteins associate with CEC and whether there is a potential effect on CETP activity. Dichotomizing CEC and CETP by low and high values showed significant positive HDL-C and ApoA-I correlation with low and total CEC (Supplemental Table 9). In contrast, oxHDL did not show any significant associations between the analyzed CEC groups. Moreover, total CETP activity results trended in the same direction of association observed earlier with the CCTA plaque characteristics and OMLs (Supplemental Table 10). Differences in TG-rich lipids among low and high oxHDL groups are presented in Supplemental Table 11.

The inverse association between the oxHDL and atherosclerotic plaque CCTA parameters observed in our analysis was apparently caused by ApoA-I lipoprotein as the main component of HDL particles. Accordingly, statistical and diagnostic significance of the specific less abundant oxidized sites of ApoA-I was less pronounced. Thus, we aimed to characterize and explore the relationship between oxidized sites and isolated oxApoA-I and coronary plaque phenotype. To pursue this aim, we identified patients with low and high oxHDL based on the 50th percentile threshold established from the whole cohort plasma oxHDL levels. Analyzed patients were matched by age, sex, and BMI with further demographic characteristics outlined in Supplemental Table 12.

A monoclonal mouse antibody (mAb), clone 7D3, produced by immunization with H_2_O_2_-oxidized ApoA-I (22) was used in our studies for oxHDL plasma measurement and immunoaffinity isolation. Although mAb 7D3 was found to be specific to both ApoA-I monomer and dimers (oxApoA-I form) (Supplemental Figure 1A), its affinity for circulating oxidized ApoA-I was relatively low. Indeed, Western blot analyses of native gels with mAb 7D3 detected bands for both ApoA-I and oxApoA-I only in 500 μL of human plasma, whereas bands for lower plasma volumes were undetectable (Supplemental Figure 1B). Moreover, native ApoA-I band detected by mAb 7D3 was less abundant in delipidated plasma as compared with the whole plasma gel (Supplemental Figure 1C). Our observation was in line with previous results, showing that different oxApoA-I forms in circulation were relatively low as compared with in tissue deposition. However, by optimizing isolation of oxHDL with mAb 7D3 in the immunoaffinity studies, we were able to purify and concentrate sufficient material for performing proteomics characterization of oxHDL (Supplemental Figure 1D).

Multiple-factor analysis revealed clear separation between high and low oxHDL groups resulting in identification of 19 isoforms among 7 peptides (Supplemental Figure 2A). ApoA-I isoforms with the most significant differences between the oxHDL groups included oxidation of methionine (Met) (M136), oxygenation of Met (M136), double oxygenation of tryptophan (Trp) (W132), and double oxygenation of Trp (W96) (Supplemental Table 13). Full liquid chromatography–mass spectrometry (LC-MS) spectrum of the identified peptides is shown in Supplemental Figure 2, B–D. In order to further elucidate the potential role of the identified oxidized residues for predicting CAD, we performed a correlation analysis of these isoforms with the CCTA plaque characteristics (Table 3). Interestingly, combined mono-oxidation of Met136 and dioxidation of Trp132 showed the most significant positive correlation with stable plaque parameters such as CAC score (*r* = 0.786; *P* < 0.001) and FB (*r* = 0.539; *P* = 0.012) in patients with high oxHDL plasma levels. However, Met136 mono-oxidation had significant association with high-risk plaque features, FFB and NB, in low oxHDL group.

## Discussion

**Figure 3:**
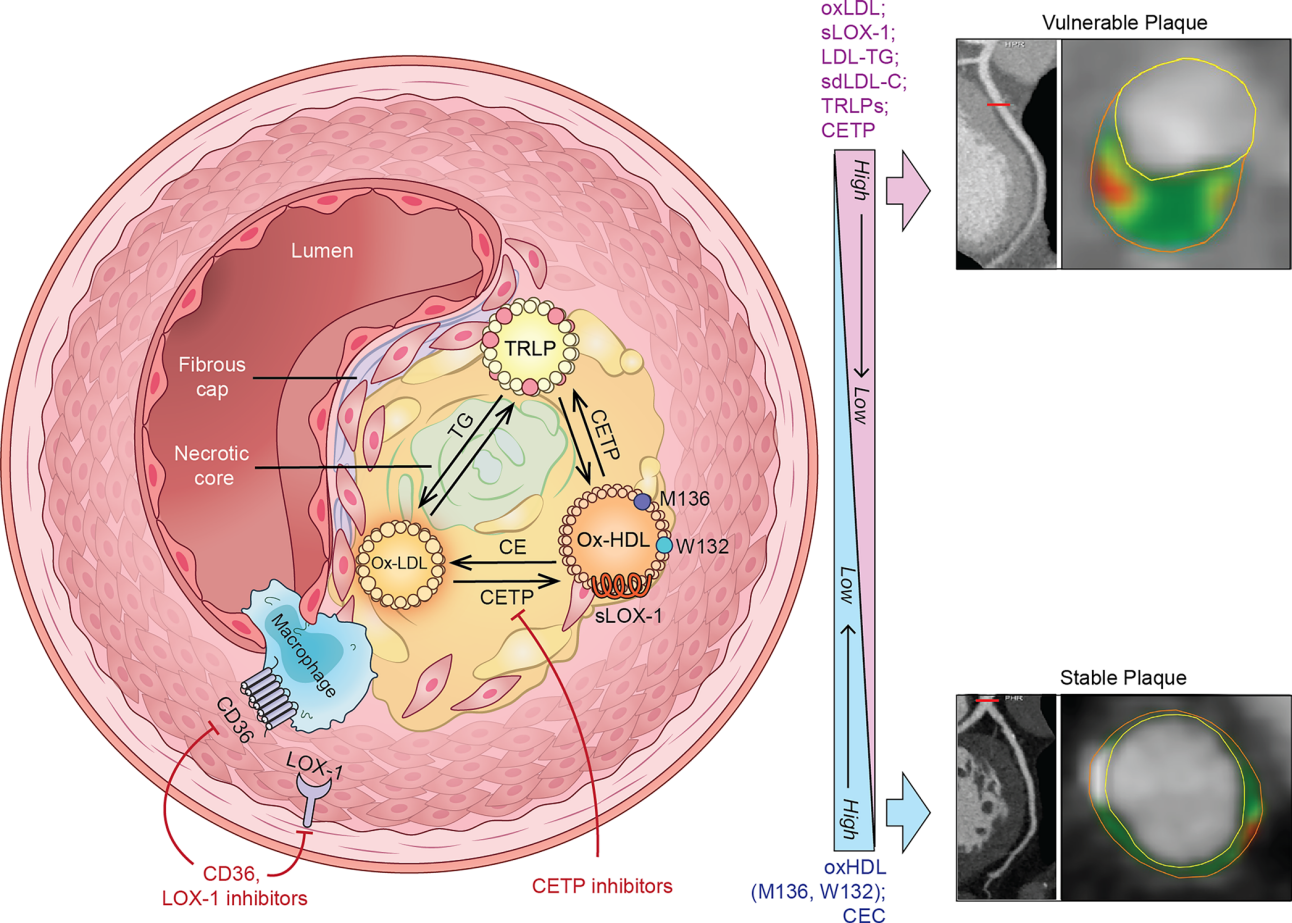
Overview of the OMLs’ association with high-risk coronary plaque phenotype and potential mechanisms of action in cardiovascular disease. Upon oxidation, both LDL (oxLDL) and HDL (oxHDL) sustain bidirectional exchange of the cholesteryl esters (CE) and TGs between each other and TG-rich lipoproteins (TRLP). This reaction is catalyzed by cholesteryl ester transfer protein (CETP), which could be altered by excessive oxidation and lipoprotein structural modifications. Indeed, oxidized ApoA-I sites (M136 and W132) may impair the reverse cholesterol transport pathway and alter cholesterol efflux capacity (CEC). Higher CETP activity results in elevated TG-rich LDL and HDL particle assembly accompanied by increased levels of TG-rich LDL (LDL-TG) and small dense LDL (sdLDL). Moreover, accumulated OMLs, specifically oxLDL, represent ligands for scavenger receptor activation on macrophages (CD36) and endothelial cells via intracellular lectin-like oxLDL receptor 1 (LOX-1). These biological reactions lead to proinflammatory cytokine and proteolytic enzyme production, which determines atherosclerotic plaque phenotype. Accumulation of OMLs, foam cells, and free lipids contributes to vulnerable plaque development characterized by lipid-rich necrotic core and thin fibrous cap. Under effective pharmacological treatment this phenotype can be switched to less rupture-prone plaque stabilized by calcium deposit and fibrous cap thickening. Indeed, newly developed therapeutics for CETP, LOX-1, and CD36 inhibition open exciting avenues for atherosclerotic coronary plaque management. Right: Representative CCTA images of atherosclerotic plaque in the left anterior descending artery of study cohort patients. Cross-sectional views demonstrate high-risk and stable plaque phenotypes. Yellow circles delineate vessel lumen, and orange circles delineate its wall. Plaque characteristics include fibrous (dark green), fibrofatty (light green), and necrotic (red) components.

Our findings indicate that circulating levels of oxidized ApoB (oxLDL) and oxidized ApoA-I (oxHDL) have different relationships with and prognostic values for high-risk coronary plaque characteristics and are increased in patients with CVD as compared with healthy individuals. Moreover, scavenger receptor–related soluble markers, such as sLOX-1, are significantly associated with vulnerable plaque phenotype. OMLs and their oxidation pathway markers may, therefore, add additional value over the use of traditional CVD risk factors. An overview of OMLs’ association with coronary plaque phenotype and potential mechanisms of action is given in Figure 3.

It has been shown that circulating oxLDL is associated with both subclinical CVD (23) and evident CVD (6), as well as a myocardial infarction (24). Indeed, accumulation of oxLDL in endothelium with macrophage uptake may contribute to atherosclerotic plaque progression and subsequent rupture (7). Although a potential role of oxLDL in the pathogenesis of atherosclerosis has been proposed, its association with coronary plaque high-risk imaging phenotype has not been fully evaluated. In our study, circulating oxLDL was significantly positively associated with plaque morphology parameters even after full adjustment for conventional risk factors. This observation replicates our previous work, which revealed a positive relationship between coronary plaque burdens and oxLDL in highly inflamed psoriasis patients (20).

Typically, negatively charged LDL particles possess a higher affinity for scavenger receptors over LDL receptors under excessive oxidative modification (24), culminating in higher circulating levels of sCD36 and sLOX-1. Indeed, the soluble form of LOX-1 activation had a strong positive association with coronary plaque burden including plaque morphological phenotype. Stratifying the cohort based on the oxLDL plasma levels was unable to capture significant differences in sCD36 and sLOX-1, which could be explained by relatively low inflammation as measured by hsCRP, less than 2.0 mg/L. As reported before, concentration of sLOX-1 and sCD36 depends on the inflammatory stimuli and becomes significantly elevated during acute CVD events, such as myocardial infarction (25). Moreover, sLOX-1 levels were shown to be predictive of fatal events beyond traditional risk factors and associated with coronary plaque progression in patients with atherosclerotic CVD (11). Recent clinical investigation of the LOX-1 pharmacological blockade in type 2 diabetes patients showed promising results that were characterized by regression of noncalcified plaque volume (26).

In contrast to oxLDL and its scavenger receptor circulating forms, oxHDL showed an inverse association with coronary plaque burden and imaging characteristics of plaque vulnerability. A recent study reported that a decrease in oxHDL, measured with the same mouse anti–oxApoA-I antibody (clone 7D3), was associated with attenuation of CAC progression in a multicenter study (19). Moreover, individuals with greater levels of oxLDL were found to have a higher coronary calcification (23), whereas in our study we did not detect the same association. The reasons for the observed discrepancies might be related to the cohort’s clinical characteristics and smaller sample size as compared with the earlier report.

Generally, dysfunction of HDL is attributed to oxidation of ApoA-I, its core structural apolipoprotein, which is targeted by either myeloperoxidase (MPO) or malondialdehyde (24). Depending on what specific amino acid residues in ApoA-I are affected by enzymatic conversion and the distribution of oxidized ApoA-I in either arterial wall or circulation, the functional properties of HDL might be strikingly different (27). Proteomic signature profiling of the circulating oxHDL in our study identified oxidized Met and Trp residues as the most commonly modified. This observation might be explained by the oxHDL concentration and, additional to Tyr, potential epitopes recognized by the 7D3 anti–oxApoA-I clone. Moreover, peptides containing double-oxygenated Met136 and Trp132 were significantly positively correlated with CAC and FB in patients with high oxHDL, whereas Met136 mono-oxidation was associated with high-risk coronary plaque features in the low oxHDL group. In line with previous reports, higher Met oxidation in ApoA-I was observed in CAD (28) and type 2 diabetes patients (29), as well as associated with impaired cholesterol efflux function (21). Interestingly, there was no correlation between oxHDL and CEC as compared with HDL-C, suggesting that the measured oxidation-modified form in our CVD cohort might be dysfunctional.

An earlier report also identified MPO-modified Trp72 of ApoA-I as the immunogenic epitope specific for isolated dysfunctional HDL, which was associated with increased CVD risk (17), whereas oxidation of Trp at 50 and 108 positions led to a loss of vasculoprotective properties of ApoA-I in samples isolated from abdominal aortic aneurysm patients (30). Antibody clone 7D3 used in our study revealed that oxidation at both Met136 and Trp132 is needed to detect the observed associations. Indeed, since ApoA-I represents the main apolipoprotein in oxidation-modified form, this might explain the inverse relationship with the coronary plaque phenotype observed in the main study cohort. Levels of oxHDL measured in psoriasis patients using the same 7D3 clone also showed an inverse relationship with the coronary plaque burden (20). As indicated previously, ApoA-I harboring oxidized residues in circulation is quite low and varies significantly between patients and disease conditions (17). ApoA-I oxidation might potentially promote its dissociation from HDL particles with subsequent diffusion into the circulation as a lipid-poor form. Thus, circulating and tissue residual fractions might be functionally and compositionally different, which depends on isolation techniques. Larger studies with less diverse populations and proper control are needed for detecting oxApoA-I presence in both circulation and atherosclerotic plaque using 7D3 and other mAb clones.

It is known that high uptake of oxLDL by monocyte-derived macrophages residing in the arterial wall leads to excessive cytokines and proteolytic enzyme release, which deteriorates atherosclerotic lesions (31). Investigation of human carotid plaques showed high oxLDL plasma and plaque levels associated with vulnerable plaque characteristics, such as thin fibrous cap and large lipid core (32), while elevated plasma sLOX-1 levels correlated with coronary plaque rupture in acute coronary syndrome patients (12). Comparison of the OMLs in predicting coronary plaque phenotype showed that oxHDL had superior clinical value in estimating necrotic and fibrofatty burdens, compared with oxLDL and sLOX-1, even after adjusting for statin treatment and hsCRP. The observed discrepancies might be related to cell-specific biological effects on lipoprotein oxidation and anatomical localization within the atherosclerotic plaque. As stated above, oxLDL is mainly processed by macrophages, which are the predominant cell types in the area surrounding necrotic cores (31). Under increased oxidation, smooth muscle cells (SMCs) can switch to synthetic phenotypes with a greater proportion of scavenger receptor expression (33). Thus, these cells not only promote fibrous cap proliferation but also contribute to cholesterol and oxidized lipid elimination alongside macrophages, the key players in reverse cholesterol transport (RCT). Indeed, SMCs can also express RCT-specific receptors, including the ATP-binding cassette transporter (ABCA1), which under excessive lipid load becomes downregulated and enhances foam cell formation (34). As a result, inefficient RCT and accumulated oxHDL may further propagate fibrofatty component expansion as seen in our CCTA analyses. Moreover, oxHDL was predictive of fibrous burden change during the 3.3 years follow-up in a limited number of patients with CVD enrolled in our study.

After stratifying the recruited patients by oxLDL plasma levels, we noticed a marked increase in TGs, accompanied by TRLPs of smaller size and LDL-TG in high oxLDL group. The same analytical approach revealed opposite and less pronounced changes in TGs and related lipoproteins under high oxHDL plasma levels. Although we observed only an insignificant trend of lower CEC in high oxLDL and no change in high oxHDL groups, the fact that CEC did not correlate with oxHDL might support its dysfunctional nature. Thus, we decided to focus on cholesteryl ester (CE) transfer between oxLDL and oxHDL mediated through CETP as a potential mechanism describing TG-related differences. Indeed, bidirectional distribution of CE among these OMLs and TRLPs represents an interesting field to explore. It has been demonstrated that oxidation of LDL enhances the CETP-mediated CE transfer rate to HDL with HDL_3_ being a relatively good CE acceptor (35) and better protector of LDL oxidation as compared with large HDL_2_ particles (16). In our study, plasma CETP activity was significantly increased in high-oxLDL patients with opposite activity in the high oxHDL group. Moreover, a strong positive association was established between total CETP activity and oxLDL with opposite negative association with oxHDL. Interestingly, high CETP activity was correlated with LDL-TG and did not show any further significant relationship with other investigated lipoproteins. Finally, CETP activity did not correlate with the CCTA plaque characteristics as compared with CEC, which showed the reverse association with coronary plaque burden.

Several limitations of our study are worth noting. First, our study was cross-sectional in nature, meaning that causality of the investigated OMLs and atherosclerotic plaque CCTA characteristics should be interpreted with caution. Second, only a minor number of patients had follow-up CCTA scans without repeated OML measurement, which limits statistical significance of the observed longitudinal data. Finally, additional validation of anti–oxApoA-I antibody is needed in more diverse and larger populations.

In summary, oxidation-modified lipoproteins and their pathway markers represent an interesting field to explore both clinically and pharmacologically. Our findings suggest that the investigated OMLs are associated with high-risk coronary plaque features and coronary plaque progression over time in patients with chronic CVD. Moreover, we provide insights into oxidation-modified HDL proteomics along with the potential contribution of CETP to increasing cholesterol ester and TG exchange between oxLDL and oxHDL. Hence, these results in addition to the recent promising clinical studies on sLOX-1 and CETP inhibition should stimulate further research in the field.

## Methods

### Study design and overview

**Figure 4:**
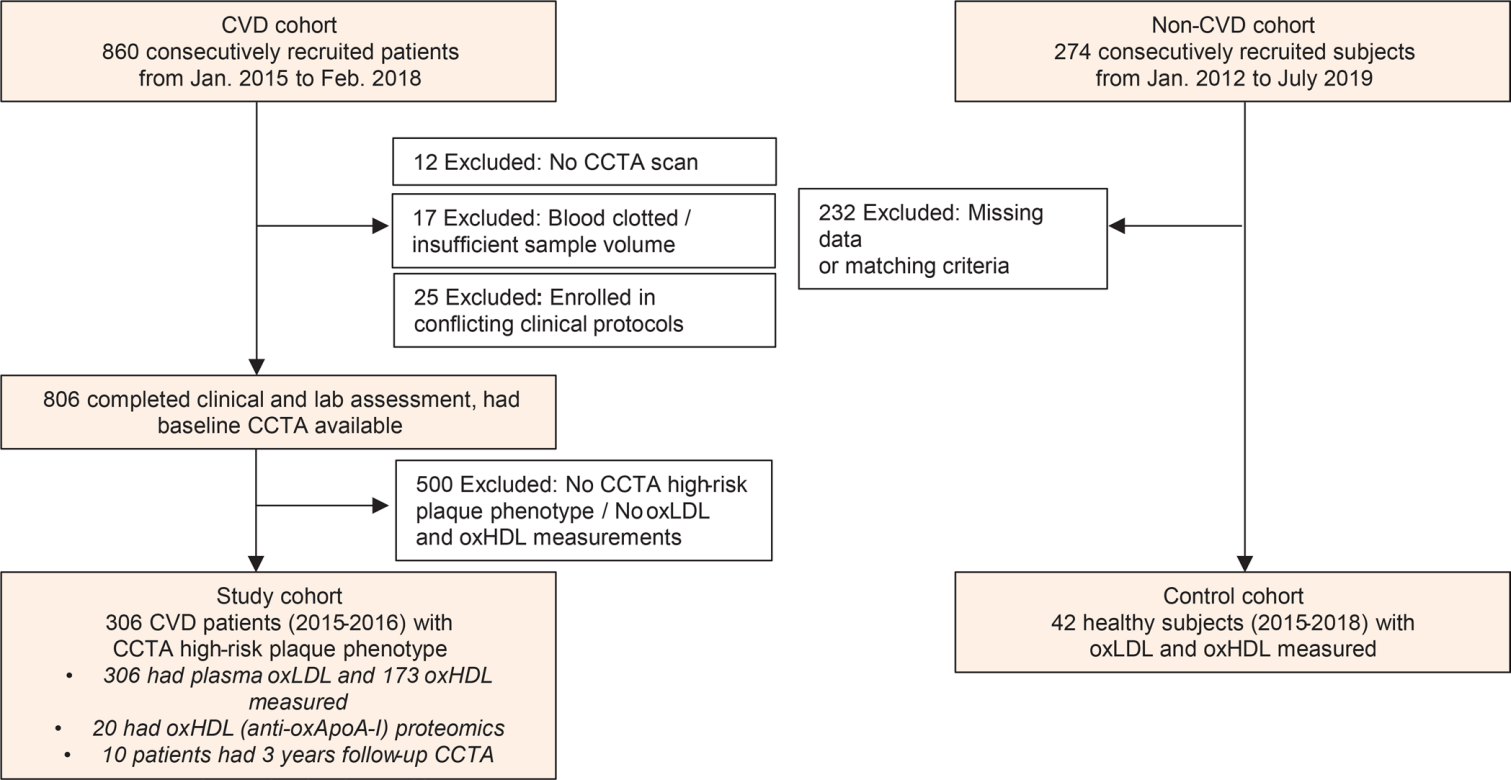
Recruitment and follow-up scheme of study participants.

A total of 860 participants with known CVD were recruited from January 2015 through February 2018 as part of an ongoing, prospective, observational study (Prospective Evaluation of New Techniques in Radiation Reduction for Cardiovascular Computed Tomographic Angiography [PREDICT]). Data analyses were reported on 306 consecutive participants who underwent additional quantitative CCTA coronary artery characterization, completed the clinical assessment, and met inclusion criteria (Figure 4). In order to prevent potential contrast-induced acute kidney injury, participants were excluded if they were pregnant or had severe renal disease (estimated glomerular filtration rate < 30 mL/min/1.73 m^2^). A complete list of inclusion/exclusion criteria can be found at ClinicalTrials.gov, NCT01621594. Retrospective deidentified demographic, clinical, and laboratory data were retrieved from medical records through the NIH clinical research repository Biomedical Translational Research Information System (BTRIS). A separate study of healthy individuals under clinical protocol C17-158 was used as a comparative control group and approved by the Ethics Committee of Jichi Medical University (Japan).

Strengthening the Reporting of Observational Studies in Epidemiology (STROBE) guidelines were followed for reporting of the findings (36).

### Biochemical measurements

#### General laboratory values and lipoprotein profile.

Peripheral blood from the enrolled participants was collected after overnight fasting in EDTA-coated tubes and centrifuged for 20 minutes at 1,500*g*. Obtained plasma was aliquoted and immediately stored at –80°C until further analysis with minimal exposure to a freeze-thaw cycle. Traditional plasma lipid parameters included total cholesterol (TC), HDL-C, and TG levels, which were measured using commercially available enzymatic methods on the Cobas 6000 analyzer (Roche Diagnostics). ApoA-I and ApoB concentrations were measured by automated turbidometric immunoassays on the Cobas 6000 analyzer. Other plasma biochemical measurements, including high-sensitivity C-reactive protein (hsCRP), were performed on a Cobas 6000 analyzer in the NIH Clinical Center. In addition, we also used homogeneous assays (Denka Seiken Co. Ltd.) for measuring direct LDL-C, TG-rich LDL (LDL-TG), and sdLDL-C as described previously (37).

CETP activity was quantified in duplicates by fluorometric CETP activity assay kit II (ab196995, Abcam).

Cellular cholesterol efflux capacity (CEC) assay was performed as previously reported. Briefly, CEC was measured in duplicate using a validated cell-based ex vivo assay involving the incubation of J774 macrophages with ApoB-depleted serum from CVD participants as previously described (38).

To further assess lipoprotein subclass profiles along with GlycA, we used the automated Vantera clinical NMR analyzer (Labcorp). The LipoProfile-4 algorithm was applied to measure the following lipoprotein subclass parameters: VLDL particle size (VLDL-Z) and number (VLDL-P); TRLP particles and the following subfractions: very small, small, medium, and large TRLP; LDL particle size (LDL-Z) and number (LDL-P), as well as their subfractions: small, medium, and large LDLP; and HDL particle size (HDL-Z) and number (HDL-P), as well as their subfractions: small HDLP (HDLP1–2), medium HDLP (HDLP3–4), and large HDLP (HDLP5–7).

#### OMLs and pathway markers.

Plasma levels of oxLDL (U/L) were measured by the quantitative commercially available ELISA kit from Mercodia (catalog 10-1143-01) with anti–ApoB-100 conformational epitope 4E6 antibody, as described previously (39). Intra-assay CV was 5.6%. Levels of oxHDL (U/mL) were detected by the quantitative in-house sandwich ELISA assay (Hoken-Kagaku West) using monoclonal mouse anti–oxApoA-I antibody clone 7D3, as described previously (22). Intra-assay CV was 4.7%. For all these assays each sample was run in duplicates and average values were used in the final analyses.

Plasma concentrations of human soluble CD36 (sCD36) were measured using human CD36 DuoSet ELISA (catalog DY1955-05, R&D Systems Inc.) in accordance with the manufacturer’s instructions. Appropriate dilutions of patient samples were measured in duplicates.

Circulating sLOX-1 was measured blindly at the NIH laboratory in plasma using an ELISA-based assay (AstraZeneca, Gaithersburg, Maryland, USA) developed with mesoscale diagnostics (MSD) (Mesoscale Diagnostics) platform. MSD high bind plates were coated with 5 μg/mL with an in-house-generated (AstraZeneca) anti–LOX-1 mAb overnight and blocked for 1 hour, and samples (25 μL/well, 1:1 dilution) were added along with recombinant human LOX-1 as standard for 2 hours. Plates were washed using MSD Tris wash buffer 4 times after each incubation step. Human LOX-1/OLR1 antibody (AF1798, R&D Systems Inc.) was sulfo-tagged using an MSD conjugation kit (R31AA-2) to generate detection antibody. Sulfo-tagged detection antibody was added and incubated for 1 hour. 2× MSD read buffer was used to read plates on the MSD machine. sLOX-1 levels from the samples were interpolated from standard curve values using MSD workbench software (Mesoscale Diagnostics). Assay was tested for matrix effects, and no interference was seen. The inter- and intra-assay variations from our analysis were less than 24%.

#### Immunopurification of plasma samples.

Before immunoaffinity isolation of oxidized ApoA-I, IgG (Fc) heavy chains were first depleted from human plasma as follows. IgG (Fc) depletion column (GWB-IGGIGY, Genway Biotech) was washed with 1 mL of 0.01% NaN_3_/PBS solution 5 times. A 500 μL aliquot of plasma samples was applied to the column and then applied for a second time followed by an additional wash with 0.01% NaN_3_/1 mL PBS. The collected through-flow fraction (1.5 mL) was then subsequently applied on anti-LDL–Sepharose 4B (supplied by MONA Ltd.) and then on an HDL depletion column (GWB-HDLIGY, Genway Biotech). The depletion columns were washed with PBS as above. The collected flow-through fraction was run 3 times through the HDL depletion column for maximum protein binding and purity. Bound proteins were stripped off from the columns by washing with 2 mL of 1× stripping buffer (0.1 M glycine/HCl, pH 2.5). After addition of 200 μL of 1 M Tris, pH 8.0, directly to the collected flow-through fraction, samples were concentrated with Amicon Ultra-4 10K (MilliporeSigma) by centrifugation at 1,700*g* for 30–40 minutes at 4°C to a final volume of 150 μL.

#### Immunoprecipitation of oxApoA-I.

After preparation of magnetic beads according to the manufacturer’s instructions (Dynabeads Protein G, Invitrogen), 20 mg monoclonal mouse anti–oxApoA-I antibody (IgG1, clone 7D3; ref. 22) was diluted in 200 μL of binding/washing buffer and resuspended with 3.0 mg of magnetic beads. After incubation with constant rotation for 60 minutes at room temperature and further washing, 150 μL of immunopurified samples from the last step was added to the magnetic beads with subsequent incubation for 45 minutes under the same conditions. After washing with 200 μL washing buffer 3 times, the Dynabeads-Ab-Ag complex was resuspended in 100 μL washing buffer and stored until further liquid chromatography–tandem mass spectrometry (LC-MS/MS) analysis.

#### Immunoblotting.

Samples of the beads were added with SDS-PAGE additionally containing 5% β-mercaptoethanol for 15 minutes at 85°C. SDS-PAGE was performed on 16% gradient gels (Novex, Invitrogen). Electrophoresis was performed as recommended by the manufacturer (Thermo Fisher Scientific). SeeBlue plus2 prestained standard (LC5925, Invitrogen) and PageRuler Plus (26619, Thermo Fisher Scientific) were used as protein size markers. The membrane was blocked with TBS, 0.1% Tween-20, 0.001% NaN_3_, and 0.1% casein for 1 hour at room temperature. Further staining for 1 hour was performed with primary mouse antibodies, anti–ApoA-I (clone 10H10, 5 μg/mL), anti–oxApoA-I (7D3; ref. 22), and goat nonimmune antibody (5 μg/mL). The secondary antibody was goat anti-mouse antibody conjugated to AP (1:10,000). Bands were visualized by application of 1-Step NBT/BCIP solution (Thermo Fisher Scientific). After gel electrophoresis, protein bands were stained for 30 minutes with Coomassie Brilliant Blue (CBB: 1 g of R-250, 400 mL of methanol, 100 mL of acetic acid, and 500 mL of double-distilled water) and then de-stained overnight (200 mL of methanol, 100 mL of acetic acid, and 700 mL of double-distilled water).

### Proteomics

#### Sample preparation.

HDL-bound beads after oxApoA-I immunoprecipitation were extracted by 100 μL of extraction buffer (6 M guanidine/HCl in 100 mM tetraethylammonium bromide [TEAB]) at 37°C for 30 minutes under constant shaking (200*g*). After magnetic separation, the remaining lysate was concentrated with Amicon 10 kDa (MilliporeSigma) to 50 μL of concentrate by spinning at 8,000*g* at 20°C for 40 minutes. After addition of 50 μL 100 mM TEAB to the residual 50 μL volume, the combined fraction was spun at 8,000*g* at 20°C for 40 minutes. In order to keep guanidine/HCl at 1 M final concentration, the above step was repeated. After measurement of protein concentration at 595 nm by Bradford assay (reagent 23246, Pierce, Thermo Fisher Scientific), 1 μg of concentrate was brought to the final volume of 50 μL with 100 mM TEAB. Reduced samples with 10 mM TCEP were incubated at 56°C, agitating for 1 hour (50*g*), followed by addition of 1 μL 500 mM TCEP stock solution. Next, samples were alkylated with iodoacetamide (A39271, Thermo Fisher Scientific) at a final concentration of 20 mM with subsequent incubation at room temperature in the dark for 45 minutes. Excess-free iodoacetamide was inactivated by addition of 1 μL of 500 mM TCEP and incubated at room temperature for 10 minutes. Trypsin (V511A, Promega) digestion was performed with an additional 4 μL of trypsin (2 μg) in 100 mM TEAB at 37°C overnight (for 40 μg proteins the ratio was 20:1). After overnight digestion, trifluoroacetic acid (TFA) was added for the final 1% TFA concertation and concentrated by SpeedVac (Thermo Fisher Scientific) at 60°C. Then peptides were dissolved in 50 μL of 0.1% formic acid in LC-MS–grade water, vortexed, and centrifuged at 6,000*g* for 1 minute. The desalting step was performed with C18 100 μL Tips (87784, Thermo Fisher Scientific). Dried peptides were resuspended in 20 μL of 2% acetonitrile/0.1% formic acid in LC-MS–grade water. Peptide digest concentrate, about 0.3 μg/μL, was vortexed for 30 seconds and centrifuged at 10,000*g* for 3 minutes. Finally, the supernatant was saved and stored at –20°C until further LC-MS/MS analysis.

#### LC-MS/MS analysis.

Desalted tryptic peptides were resuspended in 2% acetonitrile/0.01% formic acid and analyzed using nanoscale liquid chromatography–tandem mass spectrometry (nLC-MS/MS) and Ultimate 3000-nLC online coupled with an Orbitrap Lumos Tribrid mass spectrometer (Thermo Fisher Scientific). Peptides were separated on an EASY-Spray C18 column (Thermo Fisher Scientific), 75 μm by 50 cm inner diameter, 2 μm particle size, and 100 Å pore size. Separation was achieved by a 4%–32% linear gradient of acetonitrile plus 0.1% formic acid for 95 minutes. An electrospray voltage of 1.9 kV was applied to the eluent via the EASY-Spray column electrode. The Orbitrap Lumos was operated in positive ion data-dependent mode. Full-scan MS^1^ was performed in the Orbitrap with a normal precursor mass range of 375–1,500 *m/z* (mass/charge ratio) at a resolution of 120,000. The automatic gain control (AGC) target and maximum accumulation time settings were set to standard and 50 milliseconds, respectively. MS^2^ was triggered by selection of the most intense precursor ions above an intensity threshold of 2.5 × 10^4^ for higher-energy collisional dissociation (HCD)–MS^2^ fragmentation with an AGC target and maximum accumulation time settings to standard and auto, respectively. Mass filtering was performed by the quadrupole with 1.2 *m/z* transmission window, followed by HCD fragmentation in the Orbitrap at a resolution of 30,000 and collision energy of 35%. The number of MS^2^ spectra acquired between full scans was restricted to a duty cycle of 3 seconds.

#### Data analysis.

Raw data files were processed with Proteome Discoverer software (v2.4, Thermo Fisher Scientific), using Sequest HT (Thermo Fisher Scientific). The following search parameters were set: protein database UniProtKB/Swiss-Prot *Homo sapiens* (20,300 sequences release 2021_01) concatenated with reversed copies of all sequences; MS1 tolerance of 12 ppm; Orbitrap-detected MS/MS mass tolerance of 0.02 Da; enzyme specificity set as trypsin with maximum 3 missed cleavages; fixed modification of Cys (carbamidomethylation); variable modifications oxidation (M, P, W, and Y), dioxidation (W), and acetyl on N-terminus of the protein. A target decoy node was used to validate the false discovery rate of peptide spectrum matches, set to a ΔCn less than 0.05. The protein quantification was performed using Proteome Discoverer, label-free node (Minora feature detector). The area under the curve for the precursor ions was used to calculate the relative fold change between the oxidized peptides. The mass spectrometry proteomics data were deposited to the ProteomeXchange Consortium (data set identifier PXD040465) via MassIVE (UCSD), a member of the consortium (data set identifier MSV000091369).

Dimensionality reduction was applied to the epidemiological data for the 20 oxHDL samples in the group, and they were compared with oxHDL labels. Epidemiological data were processed by subsetting of the data and exclusion of artery, LDL-related, or irrelevant information. This subset of epidemiological data was processed using multiple-factor analysis to handle both categorical and quantitative metrics, with the categories separated into 13 different groups. The samples were plotted and categorized based on the coordinates generated by multiple-factor analysis. Analysis was performed using FactoMineR v2.4 (40). These categorizations were compared against the results from when the samples were categorized on the basis of their oxHDL labels.

#### Peptide isoform comparison.

Each peptide was reviewed to group together the entries separated as a result of missed cleavages. The isoforms with the same protein modifications were grouped together, and the normalized abundance values were added together on a per-sample basis. This resulted in a total of 19 peptide isoforms across the 7 peptides. Each of these isoforms was processed, and a 2-tailed *t* test was run comparing the normalized abundances between the 2 groups for each of the 4 categorizations.

#### Coronary artery imaging.

All participants underwent CCTA on the same day as the blood draw, using the same CT scanner (320-dectector row Aquilion ONE ViSION, Toshiba). Radiation exposure was in accordance with the NIH Radiation Exposure Committee guidelines. CCTA scan evaluation was done based on the CAD-RADS classification (41). All scans were initially reviewed for quality and presence of artifacts, thus making impossible a reliable qualitative and quantitative evaluation. Coronary artery burden adjusted for luminal attenuation was evaluated across each of the 3 main coronary arteries by means of semiautomated software QAngio CT (Medis) (42). Manual adjustment of inner lumen and outer vessel wall delineations was performed if needed. Total coronary artery burden, noncalcified coronary artery burden, and dense-calcified coronary artery burden indices (mm^2^) were calculated by division of total vessel plaque volume by total vessel length. Total plaque burden was defined as the sum of calcified plaque burden and noncalcified plaque burden. Noncalcified plaque subcomponents including fibrous, fibrofatty, and necrotic burdens were obtained after adaptive correction for lumen attenuation and depicted based on Hounsfield units.

CAC was evaluated as a part of normal workflow by an experienced cardiologist, using semiautomated software (SmartScore, GE Healthcare). CAC (mean total Agatston scores) was measured using electron beam tomography from 40 continuous 3-mm-thick computed tomograms (Imatron). A single, experienced radiological technologist performed scoring, blinded to clinical and laboratory data, using customized software (Imatron). Natural log-transformation of CAC scores, (ln[CAC+1]), was performed to account for the high percentage of CAC scores of 0 in all groups (43).

### Statistics

Data are presented as mean ± SD for parametric variables, median (IQR) for nonparametric variables, and number (%) for categorical variables. Skewness and kurtosis measures were considered to assess normality. Nonnormally distributed data were log-transformed to account for non-Gaussian distributions. Intergroup comparison was done by 2-tailed Student’s *t* test for parametric variables, Wilcoxon’s rank-sum test for nonparametric variables, and Pearson’s χ^2^ test for categorical variables. Spearman’s correlation testing and univariable linear regression analyses were performed to assess the potential relationship between quantitative CCTA plaque characteristics and levels of oxidized lipoproteins and related pathway markers. We modeled oxLDL and oxHDL as continuous and categorical variables, respectively. The cutoff points for the categorical variable were obtained from the distribution of both oxidized lipids in the studied population of 306 participants for oxLDL (low [<50th] and high [>50th] percentile values were <88.29 U/L [low] and >88.29 U/L [high], respectively) and 173 participants for oxHDL (low [<50th] and high [>50th] percentile values were <646.49 U/mL [low] and >646.49 U/mL [high], respectively). The cutoff point levels for low/high CEC and CETP were based on the 50th percentile values accordingly.

In the multivariable regression analysis models, we adjusted for covariates by including traditional cardiovascular risk factors, such as age, sex, current smoking, BMI, statin treatment, hsCRP, LDL-C, HDL-C, and TGs. Standardized β-coefficient along with *P* values adjusted based on the Bonferroni method to control for the type I error of multiple comparison is reported for these analyses.

To further estimate the prognostic value of the oxidized lipids for coronary artery burden, logistic regression models were used to calculate the odds ratios (ORs) and 95% CIs after dichotomizing of CCTA plaque characteristics based on median values. Multivariable logistic regression analyses were used to compare the ORs from the base model with those from the model with LDL-C, HDL-C, oxLDL, oxHDL, sCD36, and sLOX-1. The base model was adjusted for age, sex, current smoking, BMI, statin treatment, and hsCRP. Analysis was performed using Stata/IC 12.1 (StataCorp LP).

### Study approval

Study approval was granted by the National Heart, Lung, and Blood Institute and the institutional review board of Jichi Medical University (Japan) in keeping with the Declaration of Helsinki. All study participants submitted written informed consent prior to enrollment.

### Data availability

Data supporting the findings of this study are available to qualified researchers trained in human participant confidentiality protocols from the corresponding author upon request. The mass spectrometry proteomics data related to this article can be found at https://proteomecentral.proteomexchange.org/cgi/GetDataset?ID=PXD040465, hosted at ProteomeXchange Consortium as PXD040465.

## Author contributions

AVS and ATR had full access to all the data in the study and take responsibility for the integrity of the data and the accuracy of the data analysis. AVS, MYC, and ATR conceived the study concept and designed the study. AVS, CGH, AMA, EMF, JT, NP, INB, PMP, VC, SRW, ELO, and MPP acquired and analyzed the data. AVS drafted the manuscript. AVS, AMA, INB, VC, AC, TLE, KK, AVB, and ATR provided critical revisions of the manuscript. AVS and HL performed statistical analyses. AMA, AVB, and ATR provided technical guidance to AVS during the study. The study was conducted under the supervision of AVB and ATR.

## Supplementary Material

Supplemental data

Supporting data values

## Figures and Tables

**Table 1:**
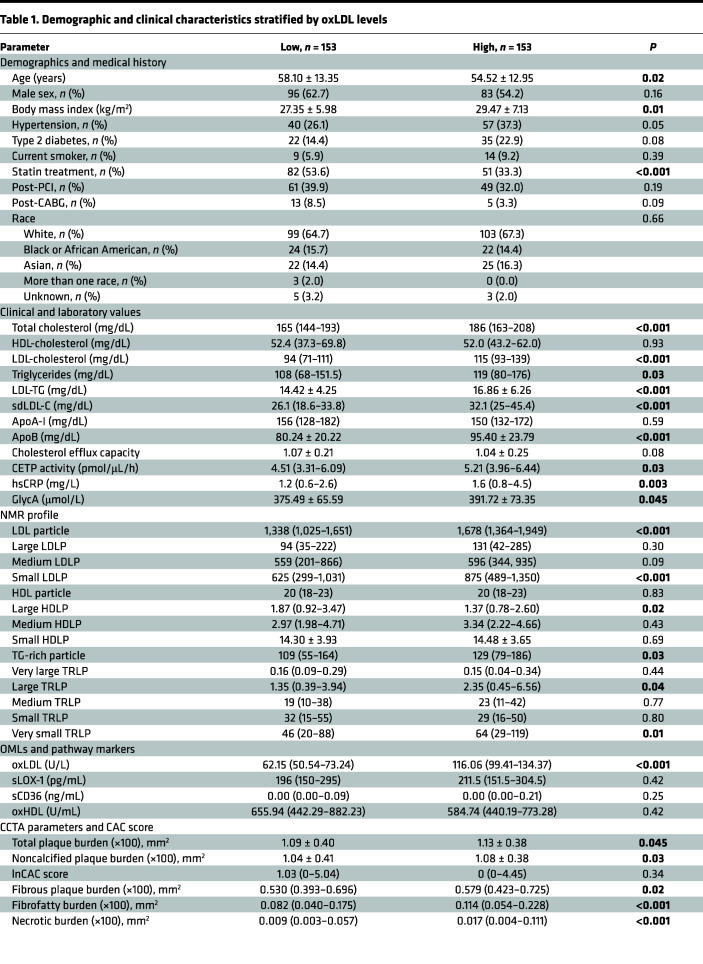
Demographic and clinical characteristics stratified by oxLDL levels

**Table 2:**
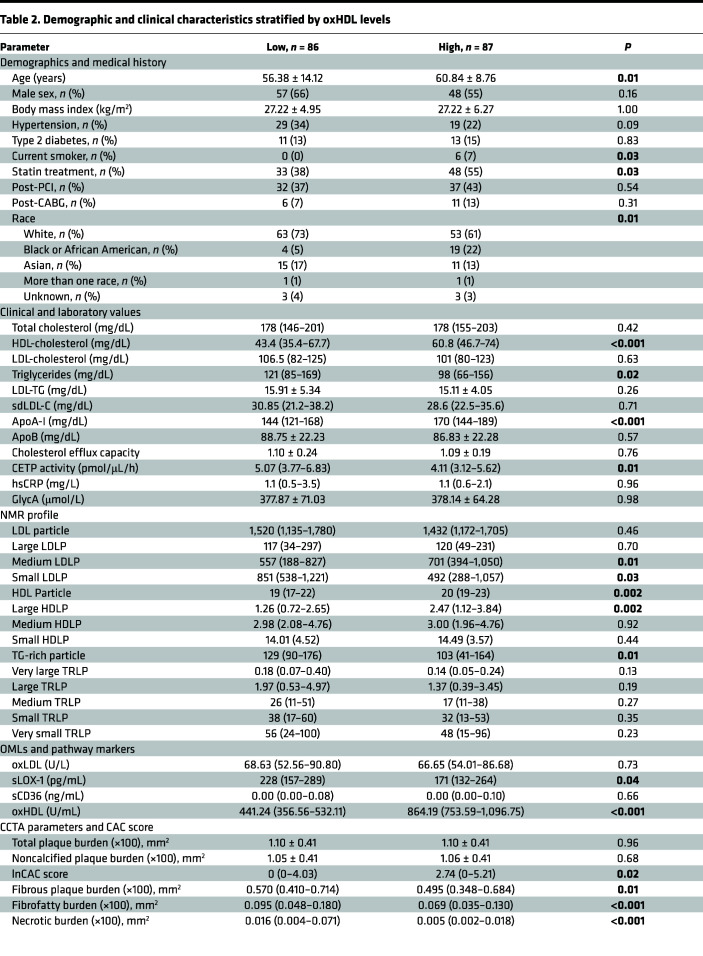
Demographic and clinical characteristics stratified by oxHDL levels

**Table 3:**
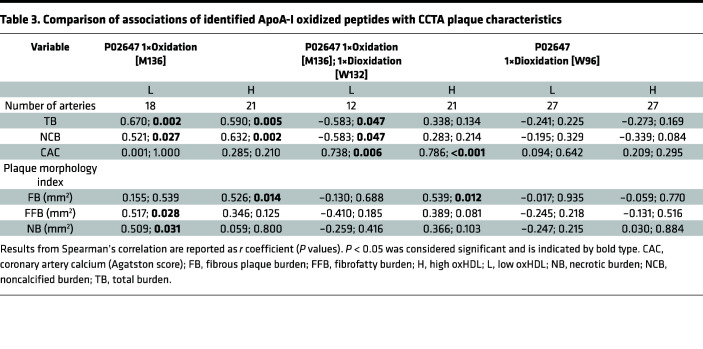
Comparison of associations of identified ApoA-I oxidized peptides with CCTA plaque characteristics

## Version 1 09/12/2023

In-Press Preview

## Version 2 10/23/2023

Electronic publication
