# Supplementary material for: Association of oxidized ApoB and oxidized ApoA-I with high-risk coronary plaque features in cardiovascular disease
Source: JCI Insight. 2023 Oct 23;8(20):e172893. doi: 10.1172/jci.insight.172893 (PMC10619497; doi:10.1172/jci.insight.172893)
Supplement: Supplemental data [file jciinsight-8-172893-s231.pdf]

Supplemental Table 1. Demographic and clinical characteristics of the study and control cohorts

Parameter	Study cohort (N = 306)	Control cohort (N = 42)	P
**Demographics and medical history**			
CAD, n (%)	280 (91.5)	0 (0.0)	**<0.001**
Age (years)	56.31 ± 13.25	51.52 ± 7.58	**0.02**
Male sex, n (%)	179 (58.5)	24 (57.1)	0.87
Body mass index (kg/m^2)	28.41 ± 6.65	23.15 ± 3.16	**<0.001**
Hypertension, n (%)	97 (31.7)	0 (0.0)	**<0.001**
Race			
White	202 (66.0)		
Black or African American	46 (15.0)		
Asian	47 (15.4)	42 (100)	**<0.001**
More than one race	3 (1.0)		
Unknown	7 (2.3)		
American Indian/Alaskan Native	1 (0.3)		
Type 2-diabetes, n (%)	57 (18.6)	0 (0.0)	**0.002**
Current smoker, n (%)	23 (7.5)	0 (0.0)	0.07
Statin treatment, n (%)	133 (43.5)	0 (0.0)	**<0.001**
Post-PCI, n (%)	110 (35.9)	N/A	1.0
Post-CABG, n (%)	18 (5.9)	N/A	1.0
**Clinical and laboratory values**			
Total cholesterol (mg/dL)	175 (153-202)	214 (187-231)	**<0.001**
HDL cholesterol (mg/ dL)	52.15 (40.5-66.9)	59.5 (50-69)	0.06
LDL cholesterol (mg/dL)	104.5 (81-125)	122 (112-147)	**<0.001**
Triglycerides (mg/dL)	114 (74-165)	96.5 (73-128)	0.08
LDL-TG (mg/dL)	15.64 ± 5.48	N/A	1.0
sdLDL-C (mg/dL)	29.3 (21.5-37.6)	N/A	1.0
ApoA-I (mg/dL)	151 (130-178)	N/A	1.0
ApoB (mg/dL)	87.84 ± 23.31	N/A	1.0
Cholesterol efflux capacity	1.06 ± 0.23	N/A	1.0
CETP activity (pmol/µL/hr)	5.10 (3.51-6.35)	N/A	1.0
hsCRP (mg/L)	1.3 (0.7-3.3)	N/A	1.0
GlycA (µmol/L)	383.61 ± 69.94	N/A	1.0
**NMR profile**			
LDL Particle	1488 (1149-1790)	N/A	1.0
HDL Particle	20 (18-23)	N/A	1.0
TG-Rich Particle	121 (69-176)	N/A	1.0
**OMLs and pathway markers**			
oxLDL (U/L)	88.29 (62.15-116.06)	49.20 (41.8-59.97)	**<0.001**
sLOX-1 (pg/mL)	204 (150.5-303.5)	N/A	1.0
sCD36 (ng/mL)	0.00 (0.00-0.16)	N/A	1.0

oxHDL (U/mL)	646.49 (442.29-864.19)	135.96 (85.1-191.16)	**<0.001**
**CCTA parameters and CAC score**			
Total plaque burden (x100), mm^2	1.11 ± 0.39	N/A	1.0
Non-calcified plaque burden (x100), mm^2	1.06 ± 0.39	N/A	1.0
lnCAC score	0 (0-4.85)	N/A	1.0
Fibrous plaque burden (x100), mm^2	0.448 (0.308-0.575)	N/A	1.0
Fibro-fatty burden (x100), mm^2	0.065 (0.034-0.108)	N/A	1.0
Necrotic burden (x100), mm^2	0.005 (0.002-0.011)	N/A	1.0

Data represented as mean ± SD or median (IQR) for parametric and non-parametric variables respectively and as n (%) for categorical variables. CAC, Agatston score; CETP, cholesteryl ester transfer protein; hs(CRP), high-sensitivity C-reactive protein; OMLs, oxidation-modified lipoproteins; oxHDL, oxidized HDL; oxLDL, oxidized LDL; sCD36, soluble CD36; sLOX-1, soluble LOX-1.

Supplemental Table 2. Association between oxLDL, lipid profile and OMLs pathway markers

**Parameter**	**oxLDL**		
	**LOW**	**HIGH**	**TOTAL**
**Clinical and laboratory values**			
Total cholesterol (mg/dL)	0.264; **0.001**	0.093; 0.255	0.320; **<0.0001**
HDL cholesterol (mg/ dL)	-0.021; 0.799	-0.047; 0.562	-0.011; 0.847
LDL cholesterol (mg/dL)	0.208; **0.01**	0.187; **0.02**	0.405; ; **<0.0001**
Triglycerides (mg/dL)	0.181; **0.03**	0.075; 0.358	0.175; **0.002**
LDL-TG (mg/dL)	0.120; 0.141	0.075; 0.360	0.242; **<0.0001**
sdLDL-C (mg/dL)	0.317; **0.0001**	0.145; 0.075	0.359; **<0.0001**
ApoA-I (mg/dL)	0.029; 0.727	0.008; 0.920	-0.020; 0.724
ApoB (mg/dL)	0.350; **<0.0001**	0.134; 0.101	0.405; **<0.0001**
Cholesterol efflux capacity	-0.070; 0.389	-0.179; **0.03**	-0.152; **0.01**
CETP activity (pmol/µL/hr)	0.003; 0.969	0.067; 0.420	0.132; **0.02**
**NMR Profile**			
LDL Particle	0.324; **0.0001**	0.176; **0.032**	0.410; **<0.0001**
L-LDLP	0.038; 0.679	-0.011; 0.899	0.064; 0.315
M-LDLP	0.214; **0.011**	-0.098; 0.262	0.126; **0.04**
S-LDLP	0.082; 0.329	0.139; 0.096	0.221; **0.0002**
HDL Particle	0.091; 0.272	0.111; 0.177	0.039; 0.500
L-HDLP	-0.125; 0.131	-0.110; 0.180	-0.175; **0.003**
M-HDLP	0.022; 0.795	0.061; 0.464	0.062; 0.288
S-HDLP	0.099; 0.230	0.137; 0.095	0.089; 0.126
TG-Rich Particle	0.209; **0.010**	0.178; **0.03**	0.206; **0.0003**
Very Large TRLP	0.113; 0.174	-0.086; 0.320	-0.034; 0.569
Large TRLP	0.089; 0.316	0.066; 0.458	0.150; **0.016**
Medium TRLP	0.066; 0.434	0.057; 0.506	0.046; 0.444
Small TRLP	0.205; **0.020**	0.025; 0.773	0.040; 0.510
Very Small TRLP	0.121; 0.160	0.213; **0.011**	0.223; **0.0002**
**OMLs and pathway markers**			
sLOX-1 (pg/mL)	-0.009; 0.912	0.185; **0.025**	0.087; 0.138
sCD36 (ng/mL)	-0.091; 0.275	0.089; 0.288	0.058; 0.320
oxHDL (U/mL)	0.101; 0.254	0.117; 0.453	0.018; 0.818

Results from Spearman correlation were reported as *r* coefficient (P values). P<0.05 considered significant. CAC, Agatston score; CETP, cholesteryl ester transfer protein; hs(CRP), high-sensitivity C-reactive protein; OMLs, oxidation-modified lipoproteins; oxHDL, oxidized HDL; oxLDL, oxidized LDL; sCD36, soluble CD36; sLOX-1, soluble LOX-1.

Supplemental Table 3. Correlation between oxLDL and lipids of interest based on statin treatment

Parameters	Non_statin (n=173)	Statin (n=133)
LDL-C (direct)	0.353; **<0.0001**	0.429; **<0.0001**
LDL-C (calculated)	0.297; **0.0001**	0.410; **<0.0001**
LDL-TG	0.297; **0.0001**	0.230; **0.01**
sdLDL-C	0.385; **<0.0001**	0.362; **<0.0001**

Results from Spearman correlation were reported as *r* coefficient (P values). $P<0.05$ considered significant.

Supplemental Table 4. Difference between HDL and LDL particles size based on statin treatment

Parameters	Non_statin (n=173)	Statin (n=133)	P
L-LDLP	139.98 (60.06-299.46)	65.78 (21.69-158.07)	**<0.001**
S-LDLP	703.57 (267.84-1256.65)	842.56 (445.84-1113.84)	0.33
L-HDLP	1.97 (0.96-3.40)	1.25 (0.70-2.40)	**<0.001**
S-HDLP	13.48 (11.35-16.13)	15.65 (13.19-17.61)	**<0.001**

Data are presented as median (IQR). P values were derived from Wilcoxon rank-sum test. $P<0.05$ considered significant.

Supplemental Table 5A. Comparison between oxLDL (n=306) and other pathway markers association with CCTA plaque characteristics

Variable	CETP	oxLDL	LDL-C	sLOX-1	sCD36	SLDL-P
**CCTA**						
**TB**	-0.013; 0.684	0.081; **0.015**	-0.005; 0.877	0.116; **0.001**	-0.018; 0.608	0.198; **<0.0001**
**NCB**	-0.004; 0.908	0.092; **0.006**	0.026; 0.450	0.092; **0.008**	0.001; 0.982	0.205; **<0.0001**
**CAC**	-0.092; **0.007**	-0.022; 0.705	-0.094; 0.102	0.024; 0.691	-0.113; **0.001**	0.127; **0.032**
**Plaque morphology index**						
**Fibrous burden (mm^2)**	0.029; 0.395	0.071; **0.036**	0.019; 0.579	0.040; 0.239	-0.011; 0.757	0.151; **<0.0001**
**Fibro-fatty burden (mm^2)**	0.032; 0.344	0.138; **<0.0001**	-0.005; 0.889	0.033; 0.321	0.019; 0.566	0.212; **<0.0001**
**Necrotic burden (mm^2)**	0.060; 0.078	0.134; **0.0001**	-0.015; 0.650	-0.006; 0.849	0.022; 0.521	0.149; **<0.0001**

Supplemental Table 5B. Comparison between oxHDL (n=173) and other pathway markers association with CCTA plaque characteristics

Variable	CEC	oxHDL	HDL-C	ApoA-I	LHDL-P
**CCTA**					
**TB**	-0.127; **0.004**	-0.029; 0.515	-0.271; **<0.0001**	-0.288; **<0.0001**	-0.270; **<0.0001**
**NCB**	-0.102; **0.022**	-0.003; 0.948	-0.227; **<0.0001**	-0.255; **<0.0001**	-0.252; **<0.0001**
**CAC**	-0.159; **0.037**	0.149; **0.049**	-0.216; **0.004**	-0.122; 0.112	-0.201; **0.009**
**Plaque morphology index**					
**Fibrous burden (mm^2)**	0.006; 0.891	-0.138; **0.002**	-0.208; **<0.0001**	-0.250; **<0.0001**	-0.179; **0.0001**
**Fibro-fatty burden (mm^2)**	-0.036; 0.411	-0.195; **<0.0001**	-0.302; **<0.0001**	-0.342; **<0.0001**	-0.271; **<0.0001**
**Necrotic burden (mm^2)**	0.034; 0.450	-0.288; **<0.0001**	-0.276; **<0.0001**	-0.307; **<0.0001**	-0.181; **0.0001**

Results from Spearman correlation were reported as *r* coefficient (P values). P<0.05 considered significant. TB, total burden; NCB, Non-calcified burden; CAC, Agatston score; FB, fibrous plaque burden; FFB, fibro-fatty burden; NB, necrotic burden

Supplemental Table 6. Multivariable adjusted linear regression analysis for oxLDL

(A)

Model	oxLDL		
	LOW	HIGH	TOTAL
	**TB**		
Adjusted for age	-0.04; 0.44	0.12; 0.02	0.06; 0.10
Adjusted for sex	0.001; 0.99	0.07; 0.12	0.08; 0.01
Adjusted for current smoking	-0.04; 0.39	0.11; 0.02	0.06; 0.10
Adjusted for BMI	-0.07; 0.13	0.03; 0.44	-0.03; 0.39
Adjusted for statin treatment	-0.03; 0.50	0.11; 0.02	0.07; 0.05
Adjusted for LDL-C	-0.02; 0.64	0.11; 0.02	0.07; 0.06
Adjusted for TGs	-0.07; 0.16	0.11; 0.02	0.05; 0.18
Adjusted for hsCRP	-0.04; 0.42	0.04; 0.35	0.01; 0.81
Adjusted for age, sex, smoking, BMI, statin treatment, LDL-C, TGs, hsCRP	-0.07; 0.14	-0.06; 0.19	-0.02; 0.50
	**NCB**		
Adjusted for age	-0.03; 0.60	0.12; 0.01	0.06; 0.10
Adjusted for sex	0.02; 0.66	0.08; 0.08	0.09; 0.005
Adjusted for current smoking	-0.02; 0.67	0.12; 0.02	0.07; 0.05
Adjusted for BMI	-0.05; 0.27	0.03; 0.42	-0.02; 0.47
Adjusted for statin treatment	-0.01; 0.79	0.12; 0.01	0.07; 0.05
Adjusted for LDL-C	-0.01; 0.79	0.11; 0.02	0.06; 0.09
Adjusted for TGs	-0.04; 0.37	0.12; 0.02	0.05; 0.11
Adjusted for hsCRP	-0.02; 0.67	0.05; 0.31	0.02; 0.66
Adjusted for age, sex, smoking, BMI, statin treatment, LDL-C, TGs, hsCRP	-0.05; 0.24	-0.05; 0.28	-0.04; 0.25
	**CAC**		
Adjusted for age	0.02; 0.83	0.10; 0.18	0.04; 0.38
Adjusted for sex	-0.03; 0.70	0.10; 0.23	-0.01; 0.90
Adjusted for current smoking	-0.04; 0.64	0.13; 0.13	-0.02; 0.72
Adjusted for BMI	-0.05; 0.59	0.11; 0.17	-0.03; 0.61
Adjusted for statin treatment	-0.04; 0.56	0.13; 0.11	0.04; 0.45
Adjusted for LDL-C	-0.0001; 0.99	0.13; 0.11	0.03; 0.64
Adjusted for TGs	-0.06; 0.46	0.13; 0.12	-0.03; 0.61
Adjusted for hsCRP	-0.04; 0.62	0.13; 0.12	-0.01; 0.90
Adjusted for age, sex, smoking, BMI, statin treatment, LDL-C, TGs, hsCRP	0.01; 0.94	0.05; 0.53	0.09; 0.11

Results from multivariable linear regression models were reported as standardized β coefficient (P values). The Bonferroni corrected P-value threshold was 0.002. TB, Total burden; NCB, Non-calcified burden; CAC, Agatston score. CAC and high-sensitivity C-reactive protein (hsCRP) were log-transformed.

(B)

**Model**	**oxLDL**		
	**LOW**	**HIGH**	**TOTAL**
		**FB**	
Adjusted for age	-0.08; 0.10	0.09; 0.047	0.04; 0.25
Adjusted for sex	-0.04; 0.37	0.06; 0.24	0.07; 0.03
Adjusted for current smoking	-0.07; 0.15	0.08; 0.09	0.05; 0.12
Adjusted for BMI	-0.09; 0.05	0.03; 0.47	0.001; 0.98
Adjusted for statin treatment	-0.07; 0.15	0.08; 0.08	0.05; 0.18
Adjusted for LDL-C	-0.08; 0.09	0.09; 0.05	0.06; 0.10
Adjusted for TGs	-0.09; 0.07	0.08; 0.09	0.05; 0.12
Adjusted for hsCRP	-0.06; 0.20	0.05; 0.30	0.04; 0.29
Adjusted for age, sex, smoking, BMI, statin treatment, LDL-C, TGs, hsCRP	-0.09; 0.05	0.01; 0.83	0.01; 0.79
		**FFB**	
Adjusted for age	-0.05; 0.26	0.23; **<0.0001**	0.11; **0.001**
Adjusted for sex	-0.01; 0.85	0.21; **<0.0001**	0.14; **<0.0001**
Adjusted for current smoking	-0.04; 0.44	0.23; **<0.0001**	0.13; **<0.0001**
Adjusted for BMI	-0.06; 0.22	0.18; **<0.0001**	0.07; 0.04
Adjusted for statin treatment	-0.03; 0.49	0.23; **<0.0001**	0.13; **<0.0001**
Adjusted for LDL-C	-0.03; 0.52	0.24; **<0.0001**	0.14; **<0.0001**
Adjusted for TGs	-0.06; 0.20	0.23; **<0.0001**	0.12; **0.001**
Adjusted for hsCRP	-0.04; 0.38	0.19; **<0.0001**	0.09; 0.01
Adjusted for age, sex, smoking, BMI, statin treatment, LDL-C, TGs, hsCRP	-0.06; 0.17	0.15; **0.001**	0.08; 0.03
		**NB**	
Adjusted for age	-0.09; **0.04**	0.23; **<0.0001**	0.12; **<0.0001**
Adjusted for sex	-0.06; 0.21	0.22; **<0.0001**	0.14; **<0.0001**
Adjusted for current smoking	-0.08; 0.09	0.23; **<0.0001**	0.13; **<0.0001**
Adjusted for BMI	-0.10; **0.03**	0.19; **<0.0001**	0.08; 0.01
Adjusted for statin treatment	-0.08; 0.08	0.23; **<0.0001**	0.14; **<0.0001**
Adjusted for LDL-C	-0.08; 0.09	0.24; **<0.0001**	0.16; **<0.0001**
Adjusted for TGs	-0.11; **0.02**	0.23; **<0.0001**	0.13; **<0.0001**
Adjusted for hsCRP	-0.09; 0.07	0.21; **<0.0001**	0.11; **0.001**
Adjusted for age, sex, smoking, BMI, statin treatment, LDL-C, TGs, hsCRP	-0.11; 0.03	0.20; **<0.0001**	0.12; **0.001**

Results from multivariable linear regression models were reported as standardized β coefficient (P values). The Bonferroni corrected P-value threshold was 0.002. CAC, Agatston score. Necrotic burden, CAC and high-sensitivity C-reactive protein (hsCRP) were log-transformed.

Supplemental Table 7. Multivariable adjusted linear regression analysis for oxHDL
(A)

**Model**	**oxHDL**		
	**LOW**	**HIGH**	**TOTAL**
	**TB**		
Adjusted for age	-0.11; 0.08	-0.05; 0.40	-0.04; 0.36
Adjusted for sex	-0.06; 0.29	0.04; 0.45	0.03; 0.51
Adjusted for current smoking	-0.10; 0.11	-0.05; 0.39	-0.04; 0.41
Adjusted for BMI	-0.05; 0.33	-0.12; 0.04	-0.06; 0.12
Adjusted for statin treatment	-0.10; 0.11	-0.05; 0.44	-0.05; 0.28
Adjusted for LDL-C	-0.10; 0.12	-0.06; 0.35	-0.05; 0.30
Adjusted for TGs	-0.04; 0.49	-0.05; 0.39	-0.01; 0.89
Adjusted for hsCRP	-0.01; 0.91	-0.11; 0.09	-0.06; 0.16
Adjusted for age, sex, smoking, BMI, statin treatment, LDL-C, TGs, hsCRP	0.08; 0.17	-0.04; 0.47	0.01; 0.78
	**NCB**		
Adjusted for age	-0.09; 0.18	-0.04; 0.53	-0.01; 0.89
Adjusted for sex	-0.06; 0.32	0.05; 0.40	0.04; 0.32
Adjusted for current smoking	-0.10; 0.12	-0.05; 0.47	-0.02; 0.64
Adjusted for BMI	-0.05; 0.40	-0.12; 0.05	-0.05; 0.23
Adjusted for statin treatment	-0.10; 0.12	-0.04; 0.58	-0.03; 0.58
Adjusted for LDL-C	-0.10; 0.13	-0.05; 0.46	-0.03; 0.52
Adjusted for TGs	-0.04; 0.50	-0.05; 0.48	0.01; 0.83
Adjusted for hsCRP	-0.003; 0.96	-0.11; 0.10	-0.04; 0.33
Adjusted for age, sex, smoking, BMI, statin treatment, LDL-C, TGs, hsCRP	0.09; 0.10	-0.04; 0.53	0.04; 0.28
	**CAC**		
Adjusted for age	-0.13; 0.16	0.02; 0.83	0.03; 0.71
Adjusted for sex	0.02; 0.83	0.08; 0.46	0.18; 0.02
Adjusted for current smoking	-0.01; 0.92	0.05; 0.66	0.14; 0.08
Adjusted for BMI	-0.01; 0.93	0.05; 0.69	0.14; 0.07
Adjusted for statin treatment	-0.01; 0.90	0.02; 0.83	0.08; 0.25
Adjusted for LDL-C	0.003; 0.98	0.04; 0.70	0.14; 0.07
Adjusted for TGs	0.002; 0.99	0.06; 0.59	0.15; 0.05
Adjusted for hsCRP	-0.02; 0.89	0.06; 0.64	0.12; 0.12
Adjusted for age, sex, smoking, BMI, statin treatment, LDL-C, TGs, hsCRP	-0.06; 0.48	0.03; 0.80	0.02; 0.74

Results from multivariable linear regression models were reported as standardized β coefficient (P values). The Bonferroni corrected P-value threshold was 0.002. TB, Total burden; NCB, Non-calcified burden; CAC, Agatston score; high-sensitivity C-reactive protein (hsCRP); oxHDL, oxidized HDL. CAC, oxHDL and hsCRP were log-transformed.

(B)

**Model**	**oxHDL**		
	**LOW**	**HIGH**	**TOTAL**
	**FB**		
Adjusted for age	-.03; 0.68	-0.12; 0.07	-0.10; 0.02
Adjusted for sex	-.02; 0.80	-0.04; 0.50	-0.07; 0.10
Adjusted for current smoking	-.05; 0.45	-0.11; 0.07	-0.12; 0.01
Adjusted for BMI	-.02; 0.78	-0.17; 0.01	-0.14; **0.001**
Adjusted for statin treatment	-.05; 0.45	-0.11; 0.08	-0.12; 0.01
Adjusted for LDL-C	-.05; 0.47	-0.12; 0.06	-0.13; 0.004
Adjusted for TGs	-.02; 0.80	-0.12; 0.07	-0.10; 0.02
Adjusted for hsCRP	.01; 0.93	-0.16; 0.01	-0.13; 0.003
Adjusted for age, sex, smoking, BMI, statin treatment, LDL-C, TGs, hsCRP	.09; 0.17	-0.12; 0.06	-0.06; 0.16
	**FFB**		
Adjusted for age	-0.18; 0.01	-0.10; 0.10	-0.18; **<0.0001**
Adjusted for sex	-0.18; 0.01	-0.04; 0.55	-0.16; **<0.0001**
Adjusted for current smoking	-0.20; **0.002**	-0.10; 0.11	-0.19; **<0.0001**
Adjusted for BMI	-0.16; 0.01	-0.13; 0.04	-0.21; **<0.0001**
Adjusted for statin treatment	-0.20; **0.002**	-0.10; 0.10	-0.20; **<0.0001**
Adjusted for LDL-C	-0.19; **0.002**	-0.11; 0.07	-0.20; **<0.0001**
Adjusted for TGs	-0.17; 0.01	-0.10; 0.10	-0.18; **<0.0001**
Adjusted for hsCRP	-0.13; 0.03	-0.12; 0.09	-0.21; **<0.0001**
Adjusted for age, sex, smoking, BMI, statin treatment, LDL-C, TGs, hsCRP	-0.06; 0.34	-0.04; 0.51	-0.14; 0.003
	**NB**		
Adjusted for age	-0.17; 0.01	-0.12; 0.06	-0.27; **<0.0001**
Adjusted for sex	-0.16; 0.01	-0.05; 0.43	-0.23; **<0.0001**
Adjusted for current smoking	-0.18; 0.004	-0.12; 0.06	-0.27; **<0.0001**
Adjusted for BMI	-0.16; 0.01	-0.15; 0.02	-0.29; **<0.0001**
Adjusted for statin treatment	-0.18; 0.01	-0.13; 0.05	-0.29; **<0.0001**
Adjusted for LDL-C	-0.17; 0.01	-0.13; 0.05	-0.28; **<0.0001**
Adjusted for TGs	-0.17; 0.01	-0.12; 0.06	-0.27; **<0.0001**
Adjusted for hsCRP	-0.12; 0.06	-0.14; 0.04	-0.28; **<0.0001**
Adjusted for age, sex, smoking, BMI, statin treatment, LDL-C, TGs, hsCRP	-0.07; 0.28	-0.08; 0.21	-0.25; **<0.0001**

Results from multivariable linear regression models were reported as standardized β coefficient (P values). The Bonferroni corrected P-value threshold was 0.002. CAC; Agatston score; oxHDL, oxidized HDL. Necrotic burden, CAC, oxHDL and high-sensitivity C-reactive protein (hsCRP) were log-transformed.

Supplemental Table 8. Comparison between oxLDL and oxHDL association with CCTA plaque characteristics

Variable	oxLDL		oxHDL	
	LOW	HIGH	LOW	HIGH
**CCTA**				
**TB**	-0.03; 0.50	0.11; **0.02**	-0.10; 0.11	-0.06; 0.34
**NCB**	-0.01; 0.79	0.12; **0.01**	-0.10; 0.12	-0.05; 0.43
**CAC**	-0.04; 0.60	0.13; 0.12	-0.07; 0.53	0.015; 0.89
**Plaque morphology index**				
**Fibrous burden (mm^2)**	-0.07; 0.15	0.08; 0.08	-0.05; 0.45	-0.12; 0.06
**Fibro-fatty burden (mm^2)**	-0.03; 0.49	0.23; **<0.0001**	-0.20; **0.002**	-0.11; 0.08
**Necrotic burden (mm^2)**	-0.08; 0.08	0.23; **<0.0001**	-0.18; **0.004**	-0.12; 0.05

Results from univariable linear regression were reported as standardized β coefficient (P values). P<0.05 considered significant. TB, total burden; NCB, Non-calcified burden; CAC, Agatston score. oxHDL, necrotic burden and CAC were log-transformed.

Supplemental Table 9. Association between CEC levels and lipoproteins

Variable	CEC		
	LOW	HIGH	TOTAL
**HDL-C**	0.163; **0.045**	-0.048; 0.556	0.229; **0.0001**
**oxHDL**	0.168; 0.151	-0.114; 0.263	0.007; 0.930
**ApoA-I**	0.179; **0.028**	-0.034; 0.681	0.221; **0.0001**

Results from Spearman correlation were reported as *r* coefficient (P values). P<0.05 considered significant.

Supplemental Table 10. Association between CETP activity and lipoproteins

Variable	CETP		
	LOW	HIGH	TOTAL
**oxLDL**	0.083; 0.317	-0.062; 0.454	0.132; **0.023**
**oxHDL**	-0.217; **0.036**	-0.187; 0.102	-0.249; **0.001**
**TGs**	-0.129; 0.119	-0.072; 0.384	-0.095; 0.101
**LDL-TG**	0.036; 0.660	0.517; **0.05**	0.011; 0.847
**sdLDL-C**	-0.040; 0.627	-0.022; 0.786	0.034; 0.565
**LDLP**	0.046; 0.588	0.011; 0.900	0.068; 0.245
**TRLPs**	-0.040; 0.635	-0.046; 0.577	-0.018; 0.761

Results from Spearman correlation were reported as *r* coefficient (P values). P<0.05 considered significant. CETP, cholesteryl ester transfer protein; oxHDL, oxidized HDL; oxLDL, oxidized LDL.

Supplemental Table 11. Difference in triglycerides-rich lipids among low and high oxHDL groups

Parameters	LOW_oxHDL (N=86)	HIGH_oxHDL (N=87)	P
LDL-TG (mg/dL)	15.91 ± 5.34	15.11 ± 4.05	0.26
Triglycerides (mg/dL)	121 (85-169)	98 (66-156)	**0.02**
TG-Rich Particle	128.60 (90.10-175.65)	102.97 (41.19-163.89)	**0.01**
Very LargeTRLP	0.18 (0.07-0.40)	0.14 (0.05-0.24)	0.13
Large TRLP	1.97 (0.53-4.97)	1.37 (0.39-3.45)	0.19
Medium TRLP	25.95 (11.38-50.72)	16.75 (11.27-37.50)	0.27
Small TRLP	37.87 (16.72-60.15)	32.35 (13.42-53.31)	0.35
Very Small TRLP	55.71 (24.35-99.98)	47.92 (15.10-95.99)	0.23

Data represented as mean ± SD or median (IQR). P values were derived from 2-tailed Student t-test for parametric variables and Wilcoxon rank-sum test for non-parametric variables. P<0.05 considered significant.

Supplemental Table 12. Demographic and clinical characteristics of the study cohort stratified by oxHDL levels for the proteomics analysis

Parameter	LOW, n=10	HIGH, n=10	P
**Demographics and medical history**			
Age (years)	53.92 ± 13.91	60.47 ± 8.19	0.22
Male sex, n (%)	7 (70)	4 (40)	0.37
Body mass index (kg/m^2)	29.19 ± 5.28	25.77 ± 3.49	0.10
Hypertension, n (%)	5 (50)	1 (10)	0.14
Type 2-diabetes, n (%)	0 (0)	3 (30)	0.21
Current smoker, n (%)	0 (0)	1 (10)	1.00
Statin treatment, n (%)	4 (40)	7 (70)	0.37
Post-PCI, n (%)	0 (0)	2 (20)	0.47
Post-CABG, n (%)	1 (10)	1 (10)	1.00
Race			0.26
White, n (%)	8 (80)	5 (50)	
Black or African American, n (%)	0 (0)	3 (30)	
Asian, n (%)	2 (20)	1 (10	
More than one race, n (%)	0 (0)	0 (0)	
Unknown, n (%)	0 (0)	1 (10)	
**Clinical and laboratory values**			
Total cholesterol (mg/dL)	169.10 ± 67.31	176.10 ± 49.82	0.79
HDL cholesterol (mg/dL)	41.0 ± 20.95	64.40 ± 23.82	**0.02**
LDL cholesterol (mg/dL)	107.8 ± 57.93	99.40 ± 38.22	0.71
Triglycerides (mg/dL)	138 (111-346)	68.5 (61-91)	**0.01**
LDL-TG (mg/dL)	16.05 ± 3.63	15.98 ± 5.23	0.97
sdLDL-C (mg/dL)	36 (21.5-42.4)	29.3 (18.2-41.4)	0.50
ApoA-I (mg/dL)	112 (108-172)	176 (128-190)	**0.049**
ApoB (mg/dL)	87.30 ± 32.21	84.90 ± 23.47	0.85
ApoE-HDL-C (mg/dL)	2.75 (2.4-6.80)	5.95 (3.0-7.8)	0.09
ApoC-III in HDL (mg/dL)	4.73 (2.51-6.78)	3.69 (2.69-6.18)	0.94
Cholesterol efflux capacity	1.09 ± 0.14	1.20 ± 0.24	0.26
CETP activity (pmol/µL/hr)	4.98 (4.00-7.99)	4.47 (2.51-6.13)	0.36
hsCRP (mg/L)	4.75 (1.8-6.8)	1.05 (0.6-1.9)	**0.004**
GlycA (µmol/L)	438.97 ± 72.71	365.24 ± 64.64	**0.03**
**NMR profile**			
LDL Particle	1686 (999-1828)	1345 (1069-1716)	0.71
L-LDLP	184 (46-225)	90 (7-206)	0.53
M-LDLP	143 (64-1017)	841 (465-1094)	0.07
S-LDLP	55 (629-1490)	445 (397-857)	0.09
HDL Particle	18.88 ± 6.02	19.88 ± 3.07	0.64
L-HDLP	1.06 (0.45-1.52)	2.76 (2.09-3.50)	**0.03**

M-HDLP	3.90 (2.66- 4.86)	4.11 (2.38-5.03)	0.55
S-HDLP	13 (10-18)	13 (10-15)	0.60
TG-Rich Particle	232 (124-253)	54.32 (20.99-101.56)	**0.01**
Very Large TRLP	0.13 (0.08-0.23)	0.11 (0.05-0.19)	0.56
Large TRLP	2.51 (1.16-4.01)	1.15 (0.30-1.97)	0.15
Medium TRLP	32 (12-61)	15 (11-17)	0.14
Small TRLP	40 (28-68)	8 (4-24)	**0.04**
Very Small TRLP	125 (32-200)	24 (10-58)	**0.04**
**OMLs and pathway markers**			
oxLDL (U/L)	71.92 ± 18.35	71.44 ± 19.04	0.95
sLOX-1 (pg/mL)	249.2 ± 66.72	203.88 ± 98.17	0.26
sCD36 (ng/mL)	0.00 (0.00-0.00)	0.00 (0.00-0.00)	0.63
oxHDL (U/mL)	302.80 (281.96-330.03)	1258.38 (1031.27-1581.25)	**<0.001**
**CCTA parameters and CAC score**			
Total plaque burden (x100), mm^2	1.12 (1.02-1.39)	0.85 (0.70-1.13)	**0.004**
Non-calcified plaque burden (x100), mm^2	1.06 (0.87-1.39)	0.82 (0.68-1.13)	**0.01**
CAC score	0 (0-3.09)	5.01 (2.57-5.59)	**0.01**
Fibrous plaque burden (x100), mm^2	0.640 (0.445-0.746)	0.425 (0.326-0552)	**0.01**
Fibro-fatty burden (x100), mm^2	0.132 (0.073-0.309)	0.044 (0.026-0.100)	**0.001**
Necrotic burden (x100), mm^2	0.030 (0.007-0.167)	0.005 (0.002-0.016)	**0.002**

Data represented as mean ± SD or median (IQR) for parametric and non-parametric variables respectively and as n (%) for categorical variables. P values were derived from 2-tailed Student t-test for parametric variables, Wilcoxon rank-sum test for non-parametric variables, and Pearson's chi-square test for categorical variables. P<0.05 considered significant. CAC, Agatston score; CETP, cholesteryl ester transfer protein; hs(CRP), high-sensitivity C-reactive protein; OMLs, oxidation-modified lipoproteins; oxHDL, oxidized HDL; oxLDL, oxidized LDL; sCD36, soluble CD36; sLOX-1, soluble LOX-1.

Supplemental Table 13. Differences in the identified isoforms between high and low oxHDL groups

Sequence	Modification	P value	T Statistic
VQPYLDDFQKKWQEEMELYR	P02647 1xDioxidation [W132]	0.549	0.619
**VQPYLDDFQKKWQEEMELYR**	P02647 1xOxidation [M136]	0.061	2.288
**VQPYLDDFQKKWQEEMELYR**	P02647 1xOxidation [M136]; 1xDioxidation [W132]	0.061	2.302
VQPYLDDFQKKWQEEMELYR	P02647 1xOxidation [W132]	0.310	1.115
VQPYLDDFQKK	P02647 2xOxidation [P123(100); Y124(100)]	0.430	-0.816
WQEEMELYR	P02647 1xDioxidation [W132]	0.835	0.212
WQEEMELYR	P02647 1xOxidation [M136]	0.509	-0.675
WQEEMELYR	P02647 1xOxidation [M136]; 1xDioxidation [W132]	0.766	-0.305
WQEEMELYR	P02647 1xOxidation [W132]	0.311	-1.054
WQEEMELYR	P02647 2xOxidation [W132; M136]	0.804	0.252
THLAPYSDELR	P02647 2xOxidation [P189(100); Y190(100)]	0.713	-0.374
QGLLPVLESFK	P02647 1xOxidation [P244(100)]	0.273	-1.156
VSFLSALEEYTK	P02647 1xOxidation [Y260(100)]	0.584	-0.578
**EQLGPVTQEFWDNLEK**	P02647 1xDioxidation [W96]	0.057	-2.145
EQLGPVTQEFWDNLEK	P02647 1xOxidation [W96]	0.785	-0.278
EQLGPVTQEFWDNLEK	P02647 1xOxidation [M110]; 1xDioxidation [W96]	0.800	-0.274
EQLGPVTQEFWDNLEK	P02647 1xOxidation [M110]	0.469	0.755
LLDNWDSVTSTFSK	P02647 1xDioxidation [W74]	0.452	-0.771
LLDNWDSVTSTFSK	P02647 1xOxidation [W74]	0.759	-0.313

P values were derived from the two-tailed T-Test with a P<0.1 considered significant.

Supplemental Figure 1. Characterization of human plasma fractions isolated with Protein G

A

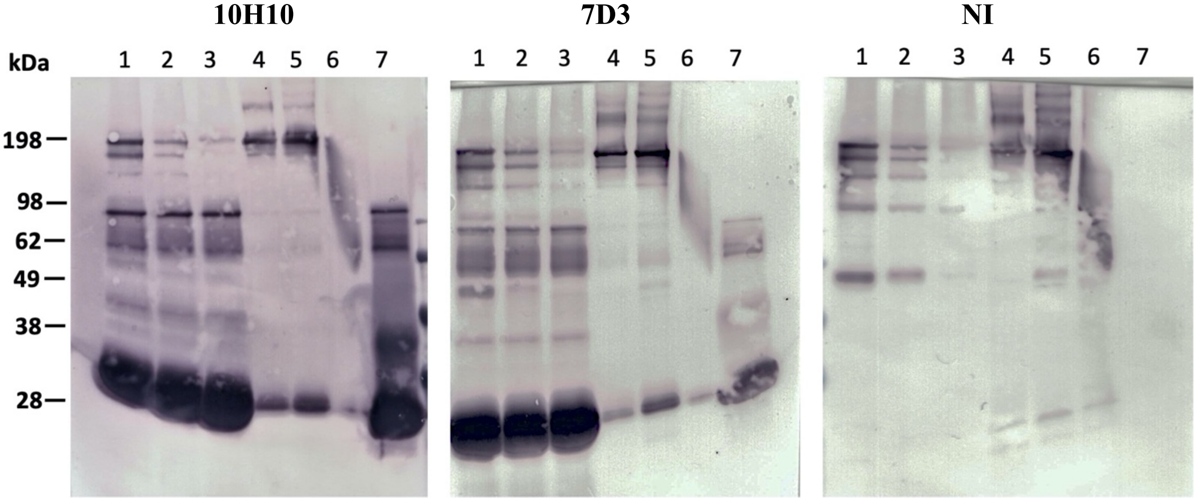

Lane 1: flow through after incubation with 10H10
Lane 2: flow through after incubation with 7D3
Lane 3: flow through after incubation with NI
Lane 4: magnetic beads after incubation with 10H10
Lane 5: magnetic beads after incubation with 7D3
Lane 6: magnetic beads after incubation with NI
Lane 7: HDL (from ultracentrifugation)

B

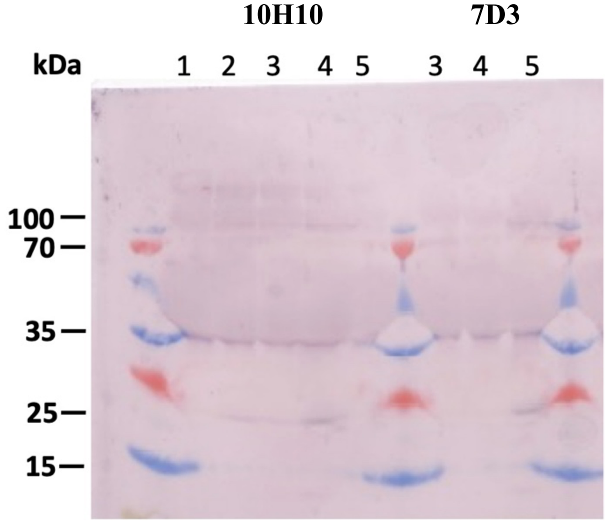

Lane 1: plasma 0 ul
Lane 2: plasma 50 ul
Lane 3: plasma 100 ul
Lane 4: plasma 200 ul
Lane 5: plasma 500 ul

C

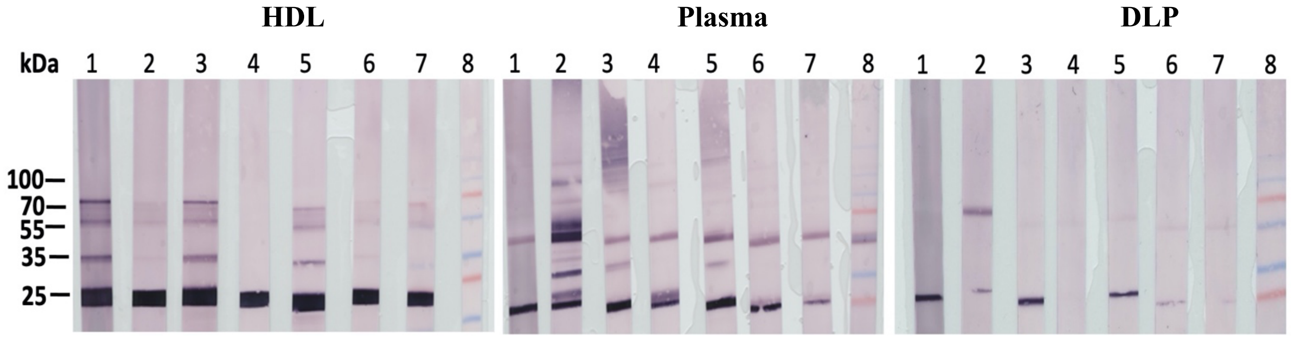

Lane 1: 10H10
Lane 2: 10A12
Lane 3: IC5
Lane 4: 10D5
Lane 5: 10E6
Lane 6: 9C11
Lane 7: 7D3
Lane 8: NI (mouse)

D

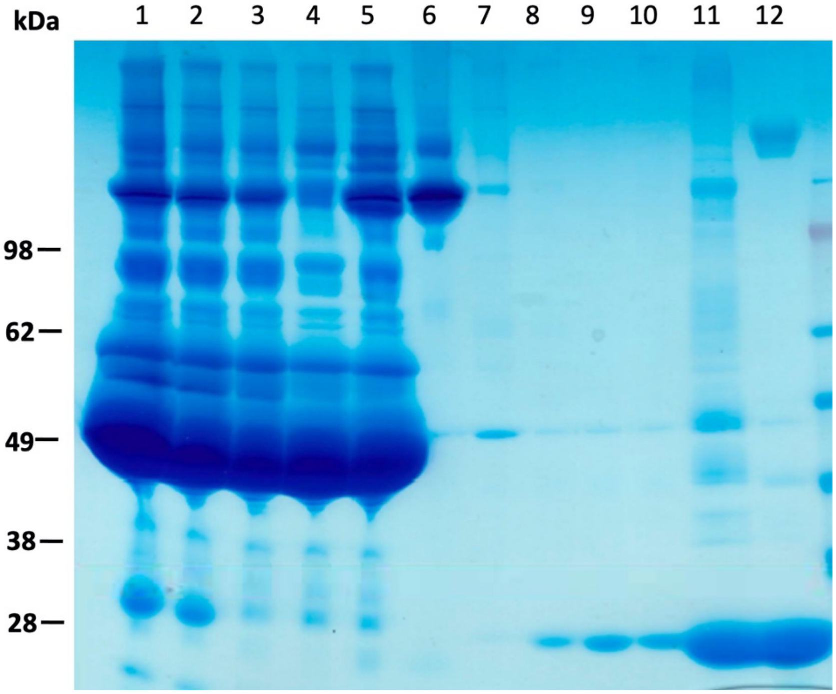

Lane 1: IgG flow through
Lane 2: ApoB flow through
Lane 3: HDL flow through column 1
Lane 4: HDL flow through column 2
Lane 5: HDL flow through column 3

Lane 6: IgG eluted
Lane 7: ApoB eluted
Lane 8: HDL column 1 eluted
Lane 9: HDL column 2 eluted
Lane 10: HDL column 3 eluted
Lane 11: HDL/oxHDL column 3 concentrated
Lane 12: ultracentrifuged HDL

(**A-B**) SDS-PAGE of human plasma stained with anti-ApoA-I (10H10), anti-oxApoA-I (7D3) and non-immune (NI) antibodies; (**C**) SDS-PAGE, detection of oxApoA-I in lipid fractions compared to different anti-ApoA-I mAbs clones; (**D**) Western blot, isolation of oxidized HDL by Protein G with anti-oxApoA-I mAb 7D3.

Supplemental Figure 2. MFA and tandem mass spectrometric analysis (LC-MS/MS) of the plasma isolated oxHDL

A

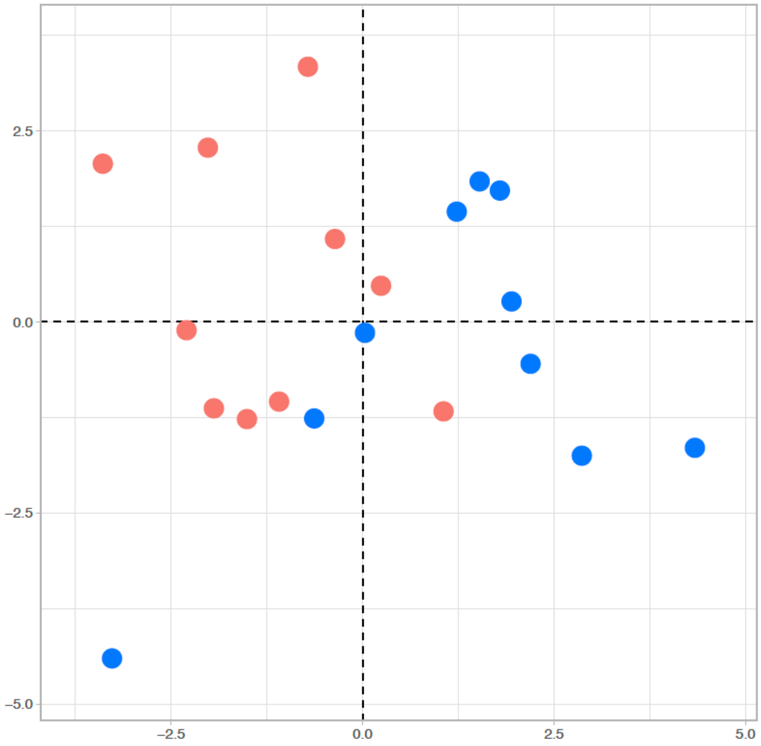

B

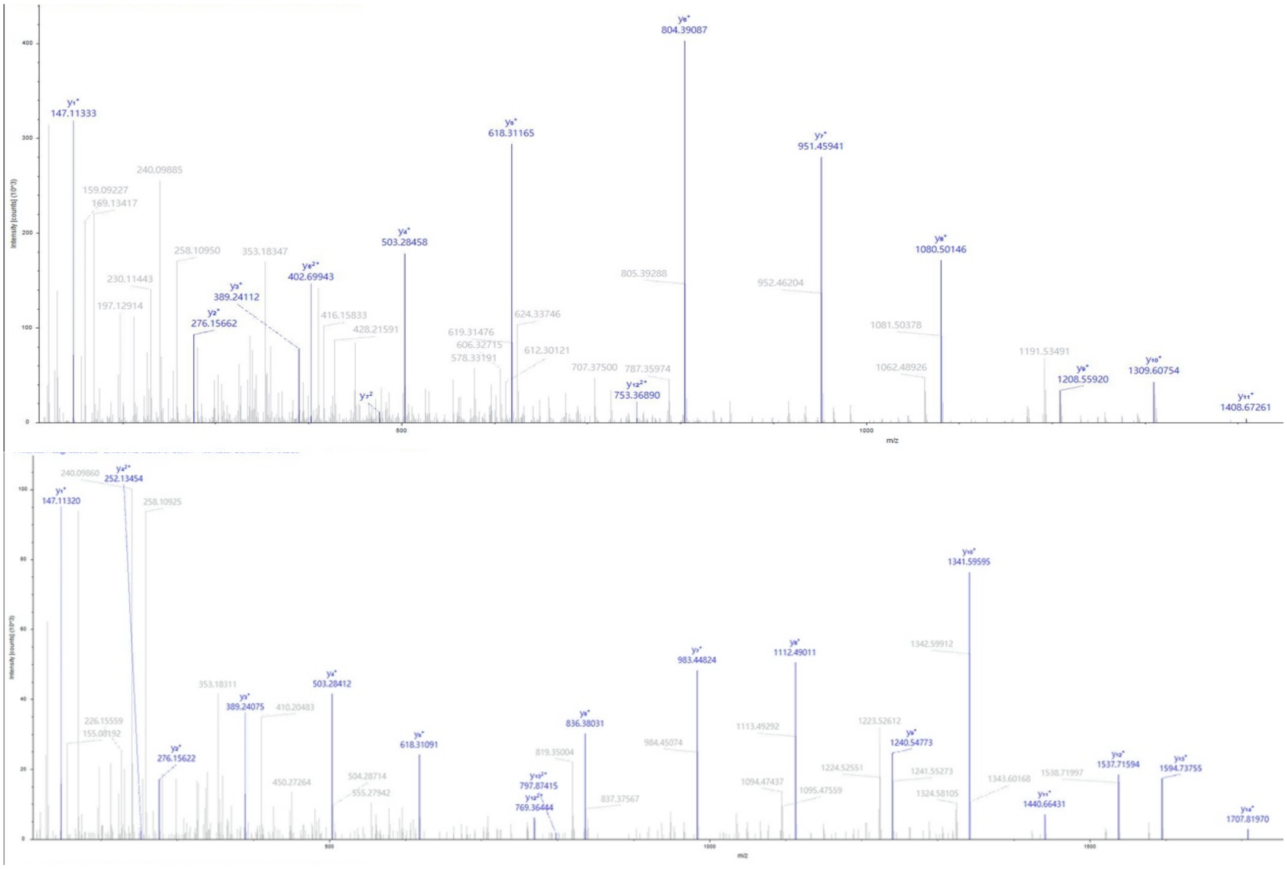
Intensity [counts] (10^3)
m/z
804.39087
147.11333
618.31165
951.45941
240.09885
159.09227
169.13417
503.28458
1080.50146
258.10950
353.18347
402.69943
230.11443
389.24112
805.39288
952.46204
197.12914
276.15662
416.15833
428.21591
624.33746
619.31476
606.32715
578.33191
612.30121
1081.50378
1191.53491
707.37500
787.35974
753.36890
1062.48926
1208.55920
1309.60754
1408.67261
240.09860
252.13454
147.11320
258.10925
1341.59595
1342.59912
983.44824
1112.49011
353.18311
389.24075
410.20483
503.28412
226.15559
155.08192
276.15622
618.31091
836.38031
819.35004
984.45074
1113.49292
1223.52612
1240.54773
1224.52551
1241.55273
1094.47437
1095.47559
1324.58105
1343.60168
1538.71997
1537.71594
1594.73755
450.27264
504.28714
555.27942
797.87415
769.36444
837.37567
1440.66431
1707.81970

C

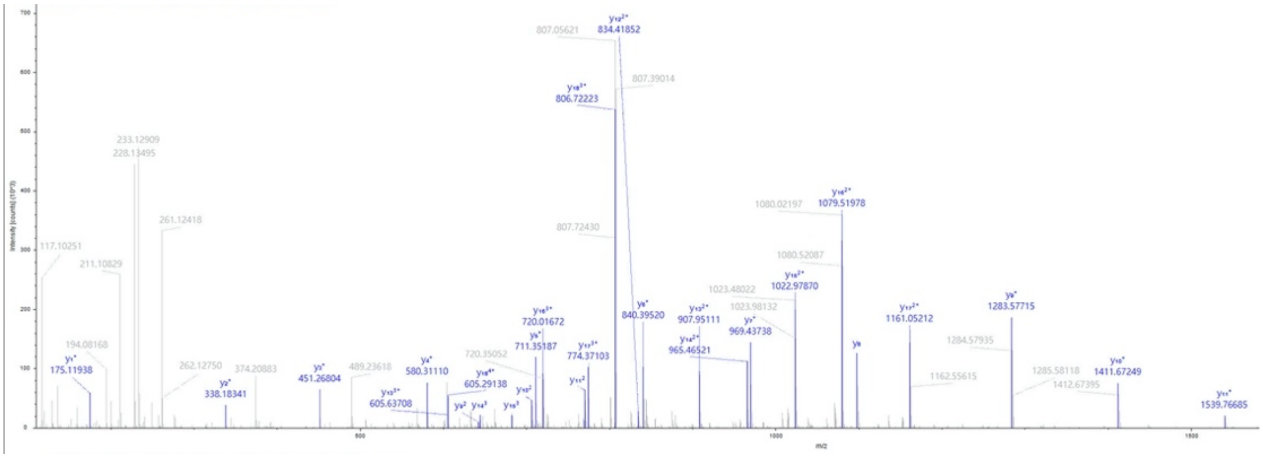
Intensity [counts] (10^3)
m/z
807.05621
834.41852
807.39014
806.72223
233.12909
228.13495
261.12418
117.10251
211.10829
1080.02197
1079.51978
807.72430
1080.52087
1023.48022
1022.97870
1023.98132
840.39520
907.95111
969.43738
965.46521
720.01672
711.35187
774.37103
1161.05212
1283.57715
1284.57935
194.08168
175.11938
262.12750
374.20883
338.18341
451.26804
489.23618
580.31110
605.29138
605.63708
720.35052
1162.55615
1285.58118
1412.67395
1411.67249
1539.76685

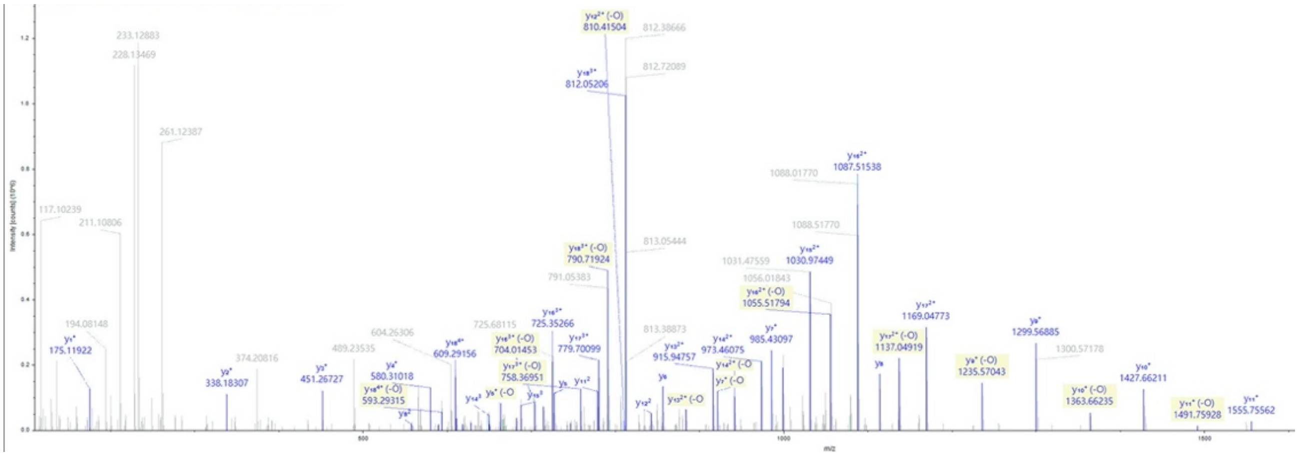

D

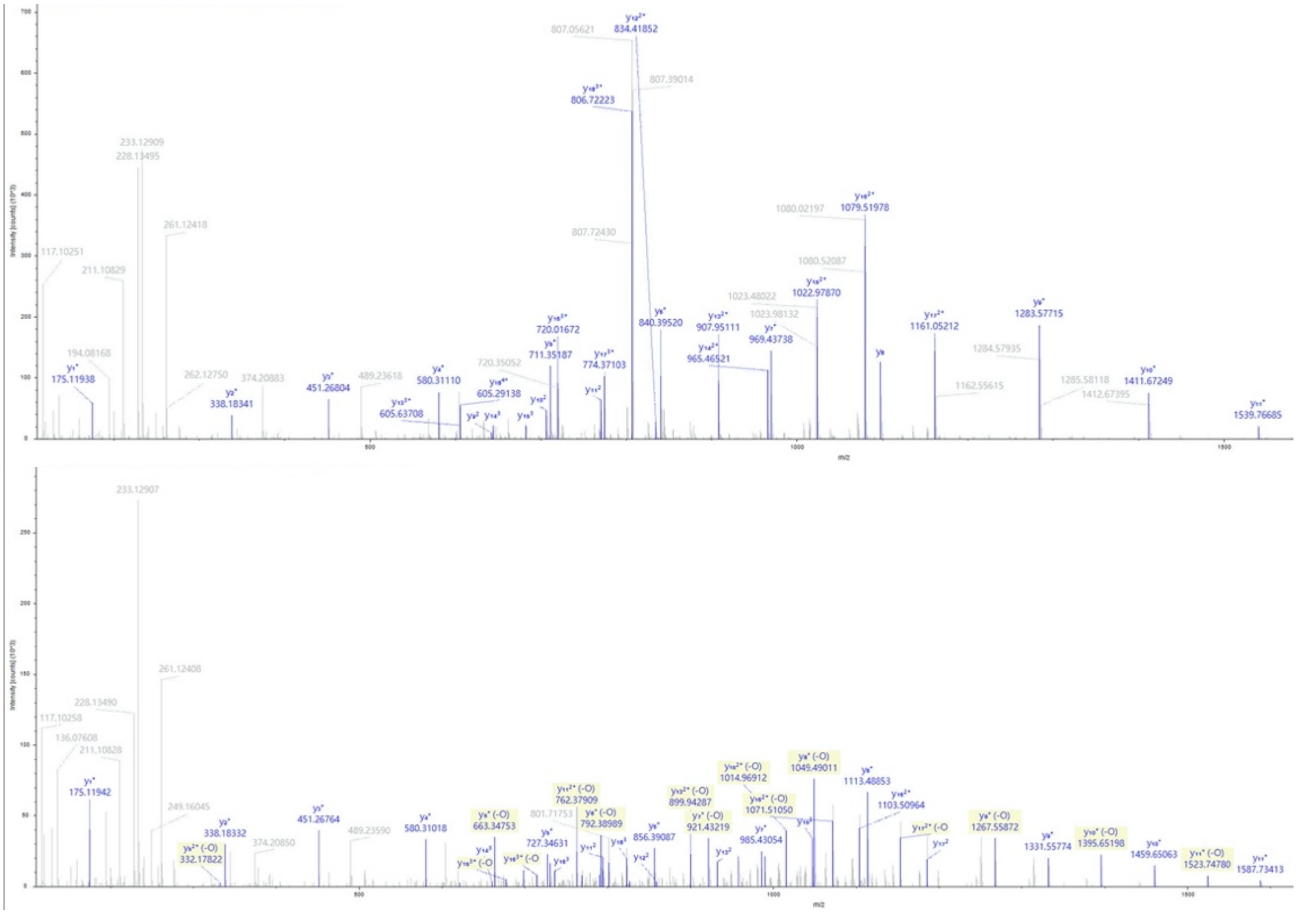

(A) 2-dimensional loading plot of the analyzed oxHDL plasma samples, red circles represent high oxHDL and blue circles are low oxHDL; MS/MS analysis identifying ions of (**B**) m/z 1932 and m/z 1964 as apoA-I peptide EQLGPVTQEFWDNLEK and the dioxidation EQLGPVTQEF$\mathbf{W}_{2ox}$DNLEK, respectively; (**C**) m/z 2645 and m/z 2661 as apoA-I peptide VQPYLDDFQKKWQEEMELYR and the oxidized VQPYLDDFQKKWQEE$\mathbf{M}_{ox}$ELYR, respectively; (**D**) m/z 2645 and m/z 2693 as apoA-I peptide VQPYLDDFQKKWQEEMELYR and the multiple oxidized VQPYLDDFQKK$\mathbf{W}_{2ox}$QEE$\mathbf{M}_{ox}$ELYR, respectively.
